# Uncovering migration systems through spatio-temporal tensor co-clustering

**DOI:** 10.1038/s41598-024-78112-z

**Published:** 2024-11-06

**Authors:** Zack W. Almquist, Tri Duc Nguyen, Mikael Sorensen, Xiao Fu, Nicholas D. Sidiropoulos

**Affiliations:** 1 Departments of Sociology and Statistics, University of Washington, Seattle, WA 98195 USA; 2 Electrical Engineering and Computer Science, Oregon State University, Corvallis, OR 97331 USA; 3 Electrical and Computer Engineering, University of Virginia, Charlottesville, VA 22904 USA

**Keywords:** Social networks, Network science, Migration, Tensors, Clustering, Dynamic clustering, Complex networks, Natural hazards

## Abstract

A central problem in the study of human mobility is that of migration systems. Typically, migration systems are defined as a set of relatively stable movements of people between two or more locations over time. While these emergent systems are expected to vary over time, they ideally contain a stable underlying structure that could be discovered empirically. There have been some notable attempts to formally or informally define migration systems. However, they have been limited by being hard to operationalize and defining migration systems in ways that ignore origin/destination aspects and fail to account for migration dynamics over time. In this work, we propose to employ *spatio-temporal tensor co-clustering*—that stems from signal processing and machine learning theory—as a novel migration system analysis tool. Tensor co-clustering is designed to cluster entities exhibiting similar patterns across multiple modalities and thus suits our purpose of analyzing spatial migration activities across time. To demonstrate its effectiveness in describing stable migration systems, we first focus on domestic migration between counties in the US from 1990 to 2018. We conduct three case studies on domestic migration, namely, (i) US Metropolitan Areas, (ii) the state of California, and (iii) Louisiana, in which the last focuses on detecting exogenous events such as Hurricane Katrina in 2005. In addition, we also examine a case study at a larger scale, using worldwide international migration data from 200 countries between 1990 and 2015. Finally, we conclude with a discussion of this approach and its limitations.

## Introduction

A central problem to the study of migration is how to define and detect migration systems^1–7^. Migration systems represent an “emergent social entity,” continually evolving and exchanging people over varying levels of spatial and temporal scales^8^. There have been some notable attempts to formally or informally define migration systems^8,9^. Still, they have been limited by either being hard to operationalize^10^ or defining migration systems as symmetric (rather than directed origin/destination of the migrant), static, or both symmetric and static. Most recently, the work by Abel et al.^8^ has employed a clustering algorithm—this is a major area of study in computational social science, social network, and network science, see for example^11–13^—which allow for directed networks^14^ to detect international migration systems over five-year aggregates of migration data; however this work only considers clustering on static snapshots which are then strung together for analysis. Using clustering methods statically focuses on differences in migration clusters rather than on finding a harmonized set of clusters over time and space.

Like previous research in the area, this article leverages the idea that one can represent migration flows as a *weighted graph* or *network*^15,16^. Here, we center on the “raw” migration data, i.e., the counts of individuals or households between two geographical units (e.g., United States and Mexico or Los Angeles County, CA and King County, WA). By representing the migration flows between such spatial units, one can employ tools from social networks^17^, network science^18,19^, and other computational social sciences^20^ to analyze this data.

Computational social science and its allied fields—*Social Network Analysis*^21^, and *Network Science*^22^—have a long history of studying network clustering and Community detection problems. Classic Community detection methods^23,24^ look for clusters of nodes in a graph or network. Primary Community detection methods in the literature include optimal modularity^25–27^, edge-betweenness^7^, leading eigenvector^28^, fast-greedy^19,29^, multi-level^30^, walktrap^31^, label propagation^32^, and infoMap^33^. However, these approaches typically do not consider the temporal modality; incorporating the time domain information in Community detection is of interest to both social and physical sciences; for example, such techniques have been used to explain Biology mechanisms^7,34^ and the group dynamics of Windsurfers^35^.

There is a well-established mathematical literature on migration dynamics, drawing on methods inspired by statistical physics. Knopoff et al.^36^ have utilized kinetic theory to model crowd behavior and migration processes, demonstrating how individual-level interactions can aggregate into large-scale patterns^37–39^. Compartment models, such as those developed by Rogers^40^ and others for multiregional demography and SIR models often solved using differential equations or stochastic methods, also play a pivotal role in understanding the distribution of populations and their transitions across different states. In the context of consensus dynamics, agent-based models have been used to explore rural-urban migration dynamics, highlighting how individual choices aggregate to influence population distributions^41^. Unlike these models that study migration from a dynamical system viewpoint, our study adopts a tensor model based on multilinear algebra. The tensor model captures the migration data’s cross-domain dependence over space and time, offering a distinct perspective.

Many network problems, such as international or domestic migration, are dynamic in nature, and a method that considers this property is preferred. There has been growing interest in holistically applying Community detection methods to dynamically evolving networks. Finding Communities in dynamically changing networks has primarily been done by using the classic Community detection methods to network “snapshots” or panel data and analyzing how the system has changed. For example^42^, studies the *change* of node associations in graphs that are collected sequentially, and^43^ studies the computational aspects of adapting new Community structures quickly based on previously estimated Communities. One can find a brief review of dynamic Community detection methods in this book chapter^44^.

Notably, dynamic Community detection^42–44^ centers on change in Community structure. Instead, our interest lies in discovering the *consistency* of Community structure over time. Within migration systems analysis, there has been one attempt at applying Community detection methods to international migration. This includes work using compartment models^40^ and other attempts to classify movement between geographies, such as gravity models^45^. More recently, network-based approaches have been applied in the field. Specifically, Abel et al.^8^ used the infoMap Community detection method over five-year migration flows and subsequently analyzed the change in Community structure over the observed periods. This article introduces a technique for holistically measuring the Community structure over time, focusing on stable Communities rather than differences. At the end of our results section, we compare the international migration system in Abel et al.^8^ to our method. Further, we compare the walktrap method applied to pre- and post-Hurricane Katrina to our method, where we find local clustering compared to a limited set of non-local clustering, and our method allows for overlapping clustering and a measure of significance for the Community/migration system.

In terms of methodology, we propose to employ a *spatio-temporal (ST) tensor co-clustering* method from the signal processing and machine learning literature^46,47^. Tensors are a natural format to store data having multiple modalities (e.g., the migration counts indexed by origin, destination, and time). Tensors also encode the cross-modality dependencies using the notion of *tensor rank*, a high-order generalization of the matrix rank. The ST tensor co-clustering method allows for a low tensor rank representation of a weighted spatial-temporal graph, e.g., origin-destination counts or other migration measures acquired over time. At each time point, the weighted graph is defined (in this case) by an origin-destination directed adjacency matrix where an edge represents the number of migrants from one spatial unit to another (e.g., Los Angeles County to New York County). This representation results in a data-driven migration system that meets the concept of a migration system in the literature (e.g., Massey et al.^1, p. 61]^). Every *rank-one* tensor extracted from the ST tensor co-clustering model represents a migration “Community” (e.g., collection of counties) whose members maintain a spatial interaction pattern with each other and share a similar temporal profile.

The ST tensor co-clustering—under this data definition—identifies the stable temporal clusters of the weighted graph (e.g., migrant counts from the United States and Mexico) and its temporal intensity over time (e.g., the Mexican-born population peaking in 2007 and decreasing post-2011; for attempts to estimate world migration rates, see^48,49^). To demonstrate the effectiveness of this approach, we consider two datasets and several case studies. First, we apply it to domestic migration data within the United States (US) from 1990 to 2018 and international migration data at five-year intervals between 1990 and 2015. The US Internal Revenue Service (IRS) makes publicly and freely available migration data at the state and county levels^50–52^. These data are built from address information in year-to-year tax returns, covering approximately 87% of all US households^53^. The IRS migration data represents a particularly unique and valuable set of migration data for the US^50^. The US Census Bureau uses the IRS migration data to produce state and county net migration estimates as part of its Population Estimate Program^50^. This data set is ideally suited for testing ST co-clustering methods. Our final case study is based on international migration. Specifically, we apply the ST tensor co-clustering approach to the international migration data constructed by Azose and Raftery^48,49^, and updated by Abel et al.^8^. International migration has a long history of theory on migration systems with a strong interest in empirically finding stable country clusters over time but with limited actual methods and applications. The ST tensor co-clustering method is again uniquely suited for this task.

**Migration Systems:** The attempt to capture the persistent interchange of people between places over time has been referred to in the literature as migration systems^1, p. 61^; according to Massey et al.^1, p. 61^: “[t]he end result is a set of relatively stable exchanges of people between [places ... yielding an identifiable geographic structure that persists across space and time.” In particular, these systems are expected to be sustained over time, emergent, and vary by spatial and temporal scales^2^, making them naturally representable by mathematical graphs or networks^3^. These systems should be expected to exist at the international and local levels^8^ as a hierarchical process. In this work, we will look to operationalize this concept of a migration system. Through the ST tensor co-clustering algorithm, we aim to find stable spatiotemporal “systems” in the United States’ internal migration and the international migration estimates from 1990 to 2015^48^.

**Spatial-temporal tensor co-clustering for migration systems:** The ST tensor co-clustering approach takes a three-way array as its input. The tensor has a size of $I \times I \times K$, where *I* is the number of geographical entities (e.g., counties, cities, and countries) and *K* is the number of temporal samples (e.g., years or months). The tensor is represented using the notation $\mathcal{X}\in \mathbb {R}^{I\times I\times K}$. Every entry of $\mathcal{X}$ has three coordinates. For example, in the IRS migration data we analyze in this work, the entry $\mathcal{X}(i,j,k)$ represents the number of migrants moving from county *i* to county *j* in year *k*. It can be regarded as a natural extension of a matrix whose entries only have two coordinates. When fixing *k*, the matrix (or the *k*th “tensor slab”) $\mathcal{X}(:,:,k)\in \mathbb {R}^{I\times I}$ is the weighted graph (e.g., origin to destination counts) collected in the *k*th year. The diagonal entries of every such matrix are ignored during data analysis using an incomplete tensor decomposition technique. The reason is that the diagonal elements do not have meaning in this migration flow analysis (i.e., we do not have measurements on within-county or within-country mobility patterns). The tensor co-clustering method decomposes $\mathcal{X}$ into the summation of *F* rank-one tensors, where *F* is pre-specified (we chose this based on information decay in the model fitting process). After the co-clustering optimization algorithm converges, *F* migration Communities will be discovered (note that we use migration system and migration Community interchangeably in this work). Each Community is represented by a tuple of vectors $({\textbf{a}}_f\in \mathbb {R}^I, {\textbf{b}}_f\in \mathbb {R}^I, {\textbf{c}}_f\in \mathbb {R}^K)$. The ${\textbf{a}}_f$ vector is an origin entity indicator, where ${\textbf{a}}_f(i)$ indicates the level of involvement of entity *i* in the *f*th migration system. The ${\textbf{b}}_f$ vector is defined similarly for destination entities. The vector ${\textbf{c}}_f$ represents the temporal profile of the *f*th migration system, i.e., how active this system is each year. Furthermore, the matrix ${\textbf{a}}_f {\textbf{b}}_f^T$ represents the spatial association of origin and destination entities in the Community, while $\varvec{c}_f$ encodes temporal intensity of the association. The tuple forms a rank-one tensor $\mathcal {C}_f$ by the outer product operation, i.e.,

$$\begin{aligned}&\mathcal {C}_f = {\textbf{a}}_f \circ {\textbf{b}}_f \circ {\textbf{c}}_f =({\textbf{a}}_f{\textbf{b}}_f^T)\circ {\textbf{c}}_f^T \\&\mathcal {C}_f(i, i, k) = 0, \quad \text {for all }1 \le i \le I, 1\le k \le K, \end{aligned}$$

where $\circ$ denotes the outer product, i.e., The above can also be expressed as

$$\mathcal{C}_f(i,j,k)={\textbf{a}}_f(i){\textbf{b}}_f(j){\textbf{c}}_f(k).$$

The readers are referred to more detailed definitions of tensor operators in Ref^46^.

**Fig. 1:**
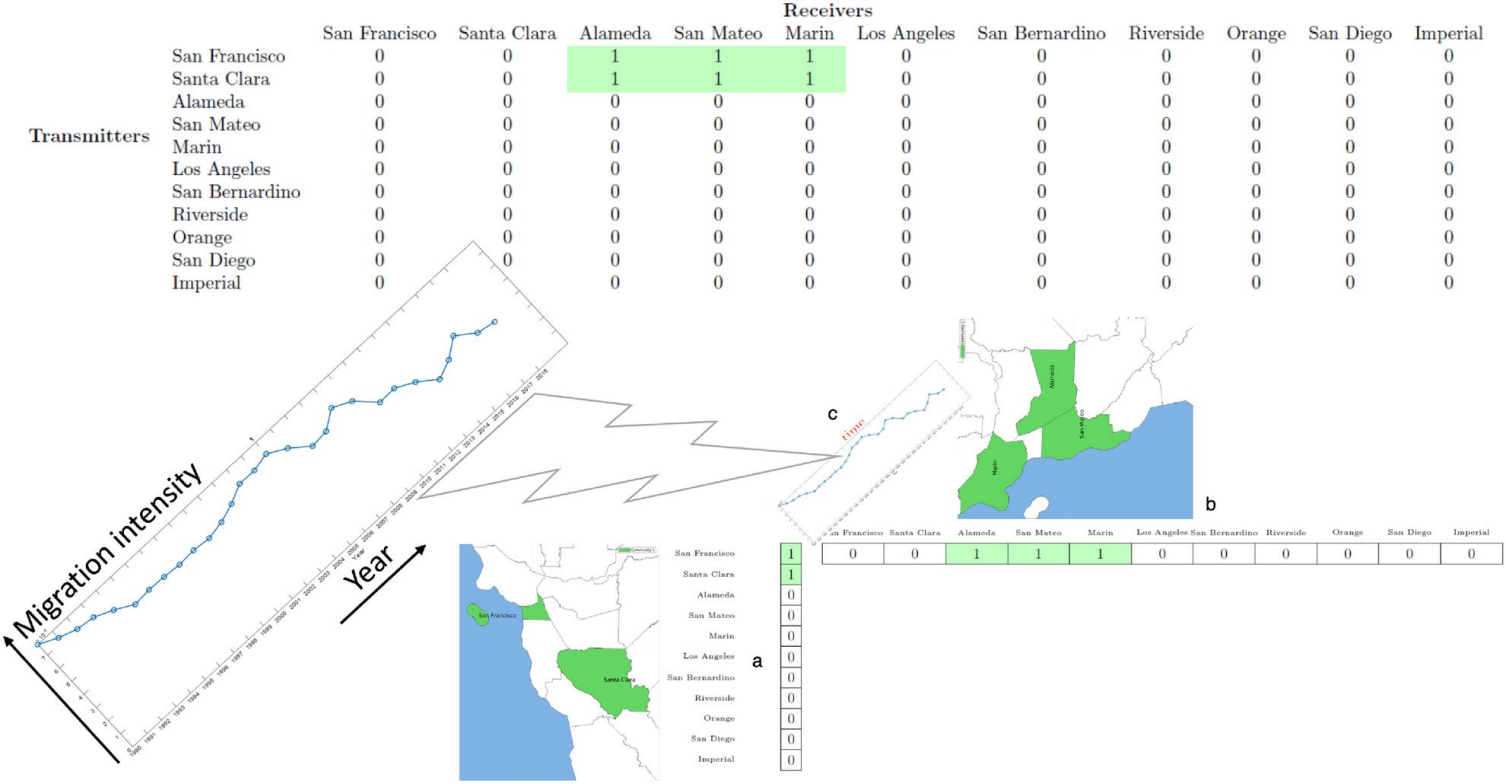
An example of the rank-one tensor-based representation of a stable migration system with its temporal profile. In this system, the origins are San Francisco and Santa Clara. Hence, ${\textbf{a}}(1)$ (San Francisco) and ${\textbf{a}}(2)$ (Santa Clara) are nonzero. The destinations are Alameda, San Mateo, and Marin, and thus, the corresponding ${\textbf{b}}(j)$’s ($j=3,4,5$) are nonzero—as shown in the lower subfigure. In addition, the top table shows ${\textbf{a}} {\textbf{b}}^T$, i.e., the spatial association of transmitters and receivers. The migration intensity is the ${\textbf{c}}$ vector, which reflects how this system’s activity level varies over the years. When multiple migration systems are simultaneously present, the associated data tensor is described by a sum of spatio-temporal rank-one terms.

This rank-one representation is exactly a stable migration system with time-varying activity levels. The rank-one tensor representation of a spatio-temporal migration system is illustrated in Fig. 1. In this system, the origins are San Francisco and Santa Clara. Hence, ${\textbf{a}}(1)$ (San Francisco) and ${\textbf{a}}(2)$ (Santa Clara) are nonzero. The destinations are Alameda, San Mateo, and Marin, and thus, the corresponding ${\textbf{b}}(j)$’s ($j=3,4,5$) are nonzero—as shown in the lower subfigure. In addition, the top table shows ${\textbf{a}} {\textbf{b}}^T$, i.e., the spatial association of transmitters and receivers. The migration intensity is the ${\textbf{c}}$ vector, which reflects how this system’s activity level varies over the years. When multiple migration systems are simultaneously present, the associated data tensor is described by a sum of spatio-temporal rank-one terms.

## Results

To illustrate the viability of using the ST tensor co-clustering method for understanding migration systems, we employ two datasets and four case studies: (i) US Metropolitan Areas, (ii) California, (iii) Louisiana with a focus on detecting exogenous events such as Hurricane Katrina in 2005 and (iv) international migration from 1990 to 2015 at five-year intervals. Case studies (i)-(iii) are conducted with IRS migration data^50^, and case study (iv) is based on international migration data from^49^.

### IRS data—US census metropolitan statistical areas

Migration scholars have often focused on the economic, social, and political impact of internal migration in the United States^54,55^. Internal migrants, unlike international migrants, are attracted to destinations other than traditional port-of-entry. The origins and destinations can respond to “pushes” and “pulls” related to environmental, political, and economic changes^56^. Frey^57^ notes that metropolitan areas are more closely aligned with the labor market or Community concept and are potentially the most appropriate geographic units for examining internal migration patterns. Here, we can employ the ST tensor co-clustering algorithm to “uncover” the stable migration systems over the last twenty or so years, and our method also allows us to observe the temporal change in migration intensity due to fluctuations (e.g., labor market) over this period.

The US Census Bureau defines 384 metropolitan statistical areas (MSAs), representing one or more counties with at least one urbanized area of 50,000 or more inhabitants. The 384 MSAs range from 20 Million (New York City–Newark–Jersey City) to 58,000 (Carson City, NV MSA). These 384 metropolitan areas represent 86% of the US population in 2020^53^. In this vein, the important question is, *can we find migration systems between these major economic regions from 1990 to 2018*? We follow up on this question by asking *how variable these Communities are over time regarding the intensity of their activities*. Based on our sensitivity analysis (see the Online Appendix), we center our analysis on six major migration systems.

The ST tensor co-clustering method provides (i) a set of migration systems, (ii) a ranking of the core migration systems, and (iii) a temporal profile of the intensity of each migration system. At its crudest, the ST tensor co-clustering method provides indicators of the associations of each US county with the six migration systems (as described in Fig. 1). The number of systems (i.e., *F* in the model) is picked by observing the residuals between the low-rank representation and the complete tensor data—see more discussions in the Appendix. The low-rank decomposition aims to find the following representation of the spatial-temporal data $\mathcal{X}$:

$$\begin{aligned} \mathcal {X} \approx \sum _{f=1}^F {\textbf{a}}_f\circ {{\textbf{b}}}_f\circ {{\textbf{c}}}_f,~ \textbf{A}\ge \varvec{0}, \textbf{B}\ge \varvec{0}, \textbf{C} \ge {\varvec{0}}, \end{aligned}$$

where $\textbf{A}=[{\textbf{a}}_1,\ldots ,{\textbf{a}}_F]$ and $\textbf{B}$ and $\textbf{C}$ are defined identically. The nonnegativity constraints are added to the factor matrices to reflect their physical meaning (i.e., the level of involvement in different migration systems for $\textbf{A}$ and $\textbf{B}$ and the activity intensity for $\textbf{C}$). The columns of $\textbf{A}, \textbf{B}$ and $\textbf{C}$ are normalized to have unit Euclidean norms (details in the Online Appendix).

**Fig. 2:**
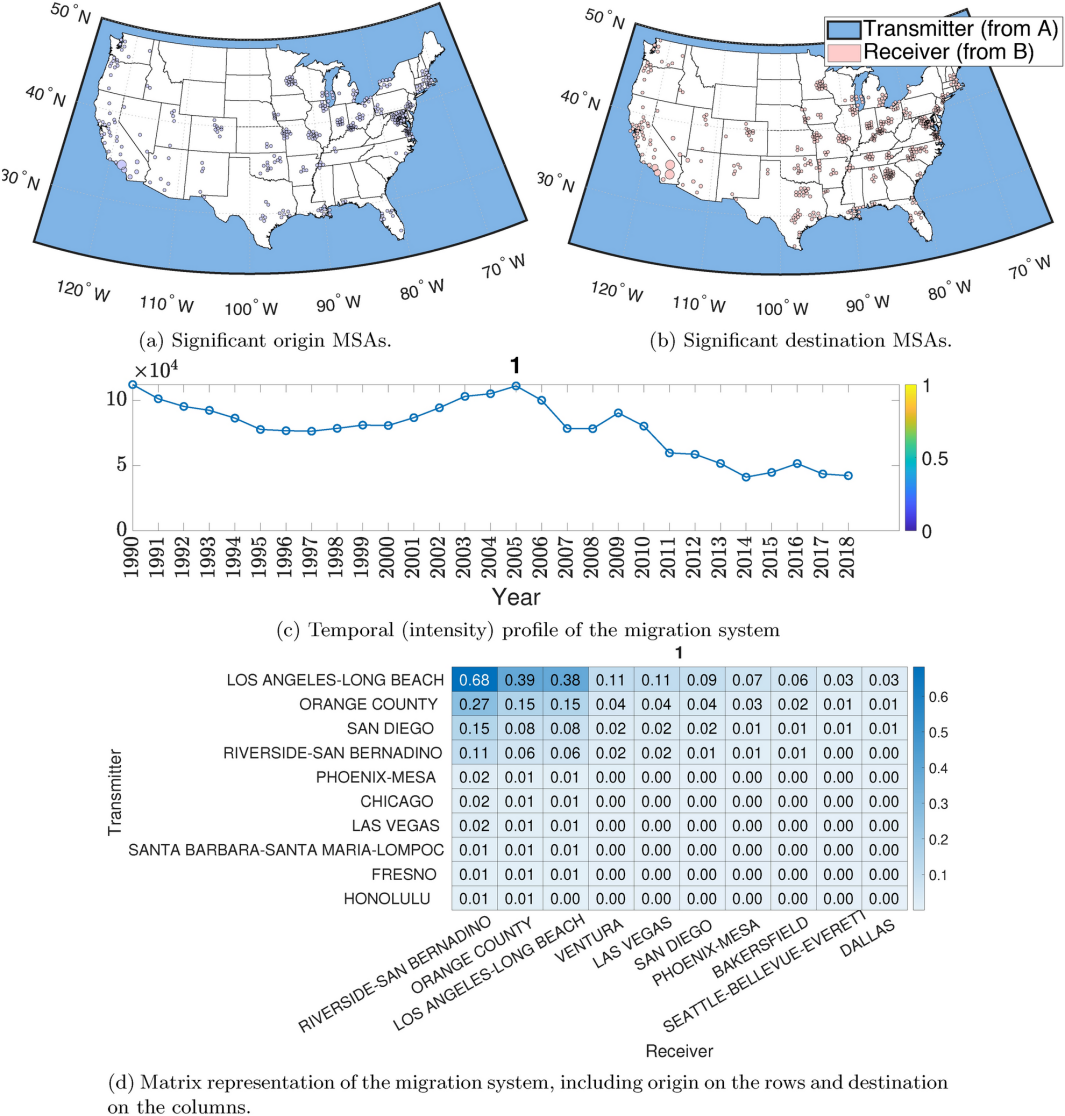
Spatial and temporal plots of US Metro origin and destination migration systems. (**a**) and (**b**) represent the visual of the significant origin and destination systems for US metropolitan areas (major cities). (**c**) contains the temporal profile of the most important component, and (**d**) is a visualization of the resulting probability matrix for the top 10 origin migration systems. In (**d**), we can see the decline in migration over the last 30 years in the US. In (**a**), (**b**), and (**c**), we see a strong sunbelt, with Texas receiving migration systems, Large Midwest and Northeast sending migration systems, and a smaller California set of sending areas.

We focus on the top five to ten counties in each migration system as the probability of a county being in the system quickly approaches approximately zero in almost all cases below this threshold (details in the Online Appendix).

#### Metropolitan migration hubs: the case of Los Angeles–Long Beach MSA

Focusing on one of the six major migration systems, the Los Angeles–Long Beach MSA, we can observe some key regional relationships that extend beyond the LA metro. See Fig. 2 where the core migration system is Los Angeles–Long Beach MSA, Orange County MSA, San Diego MSA, and Riverside–San Bernardino MSA, all in California with a secondary set of MSAs in Arizona (Phoenix-Mesa), Illinois (Chicago), and Nevada (Las Vegas).

#### The pre-housing collapse and the great migration slowdown

By engaging with the temporal intensity measures pulled out of our method, we can see major economic events like the housing slowdown and its resulting impact on migration systems in the US. Looking at Fig. 2:**(c)** time-series plot we see that this method pulls out (in an entirely data-driven way) the same qualitative story as^58^ which found that California lost most migrants to Arizona and Nevada in 2004–2005 and pre-housing collapse in 2010 a gain in migrants to Riverside–San Bernadino MSA from 2007 to 2009. We find a bump in migration intensity as US housing recovers starting in 2013/2014 (see^59^) and a decline as the housing market began to heat up in 2016. Similar to Frey^57^, and others^60^, we can observe the “great slowdown” where internal migration declines over the whole US. Notice that we can find local variation in the system, such as that of the Los Angeles–Long Beach MSA, due to the impact of the housing market. Later, we can see a rebound in the early 2000s, followed by the most recent decline.

### IRS data—California

**Fig. 3:**
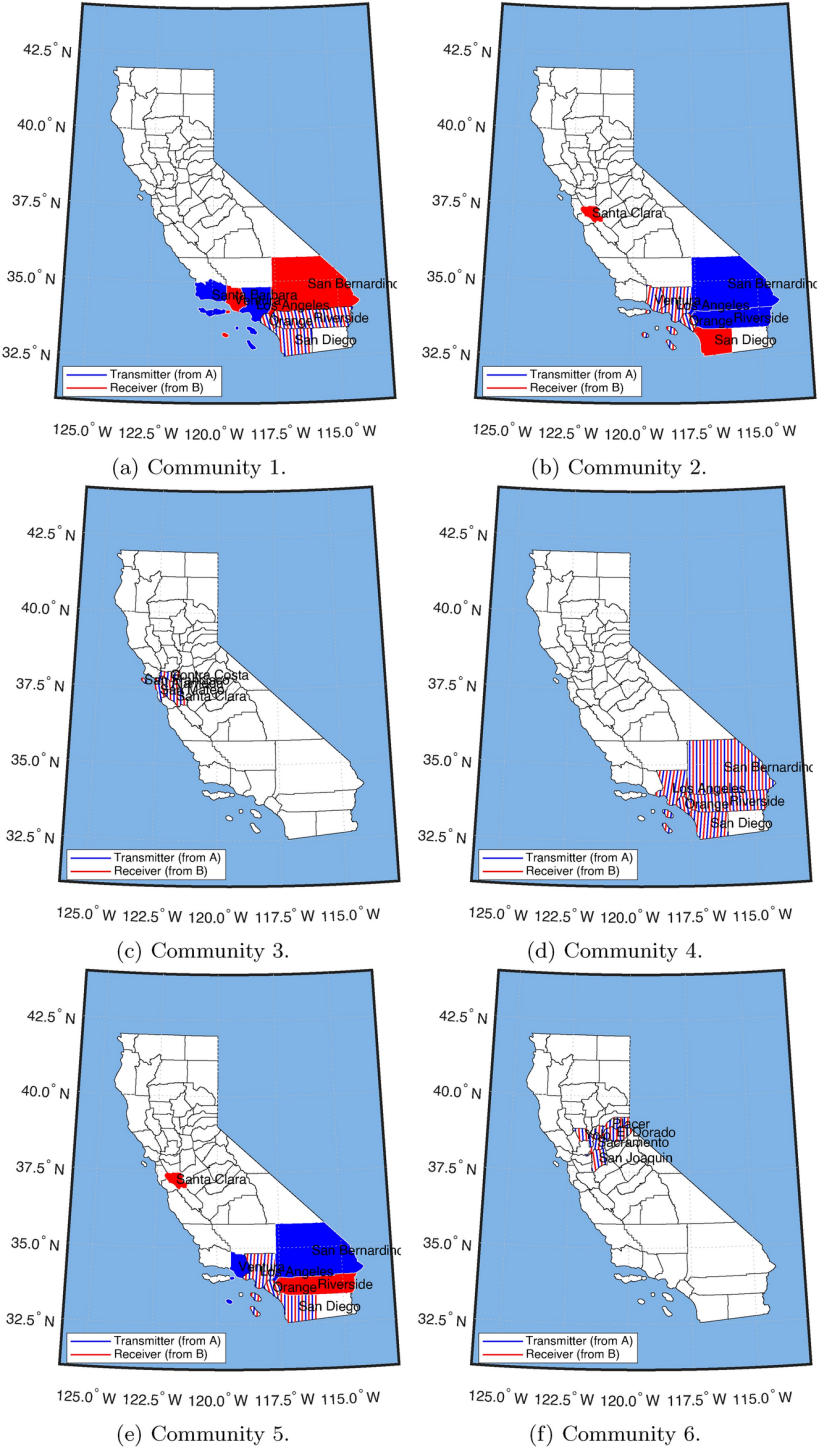
Major origin and destination migration systems (networks) for California Counties. The six tiles represent the six most important Communities with sending and receiving from 1990 to 2018. Community 1 and 4 capture the Southern California migration systems; Community 2 and 5 capture the Bay Area and Southern California migration system; Community 3 captures the Bay Area migration system; and 6 captures the Sacramento (CA capitol) migration system. Blue represents significant sending counties and red represents significant receiving counties, with purple representing counties that are significant senders and receivers.

California has been one of the most studied states for domestic migration^61^. Further, Frey^61^ Huang and Butts^62^, and others have shown that California is a high migration state with intense internal and cross-state effects. Here, we zoom into California from 1990 to 2018 and look at county-to-county migration within the state. We observe two core Communities that Southern California and Northern California define. This finding aligns with a colloquial notion of the North/South divide in California popular culture (see Fig. 3). A fundamental question in the migration systems framework is which systems represent the “core migration systems.” To understand how this method can illicit such information, we center our analysis on California because it is the largest state in the U.S., with approximately 40 million residents, and has two of the best-known regions within a state: Northern California (centered in San Francisco/Bay Area) and Southern California (centered in Los Angeles) to validate our method with.

We find a clear set of migration systems dominated by California’s Northern and Southern counties.

Our two major systems are: (i) Southern California—Communities 1 and 4 in Fig. 3a,d; and (ii) Northern California—Communities 4 and 6 in Fig. 3d,e) with Los Angeles County as the link between the two systems, Communities (b) and (e) in Fig. 3b,e.

### Southern California migration system

The Southern California system has three distinct regions: Los Angeles, the Inland Empire (Riverside and San Bernardino counties), and San Diego (Fig. 3b,d). We can see in Community 1 that the Inland Empire and San Diego are receiving migrants from the Los Angeles area—we can interpret this as people moving from higher home prices (Los Angeles County) to lower housing costs (Inland Empire and San Diego). In Community 4, reciprocal migrations occur where people move around the greater Southern California region. Further, if we look at the temporal profiles (Appendix Fig. E.11), we can see that Community 4 has been largely deactivated recently, with Community 1 being the most active. This aligns with the recent rise in housing costs and correlates with the current housing crisis and growth in homelessness^63^.

#### Northern California migration system

We can see two distinct migration systems for Northern California: one dominated by San Francisco, representing Silicon Valley (Fig. 3c), and a second one dominated by Sacramento (the capital of CA), representing the political capital of California (Fig. 3f). Next, we again look at the migration systems’ temporal profiles (Appendix Fig. E.11). We discover that in Community 3 (Appendix Fig. E.11c), the temporal intensity matches the crest of unemployment and subsequent decline in unemployment (see^64^), which reinforces the idea of the importance of labor markets on internal migration. Focusing on the temporal profiles of these migration systems, we see that the core migration system (Community 1; Fig. 3) shows the general trend known as the “Great American Migration Slowdown” (coined by Frey^58, p.1^). It is generally established that there has been a decline in internal migration since about the 1970s, with the slowdown picking up in the 1990s^53^. From the figure, we also pick up the decline in unemployment from around 2010 to 2018 (see^64^).

#### Linking northern and southern California

When we look at Communities 2 and 5 (Fig. 3b,e), we can see the link between Northern and Southern California centering around a suburb of Silicon Valley (Santa Clara County) which receives migrants from Los Angeles primarily, further, when we look at the temporal profiles, we see a big increase in movement from Southern California to the Bay Area, which correlates with the more recent tech boom.

#### Classifying the state into northern and southern California regions

**Fig. 4:**
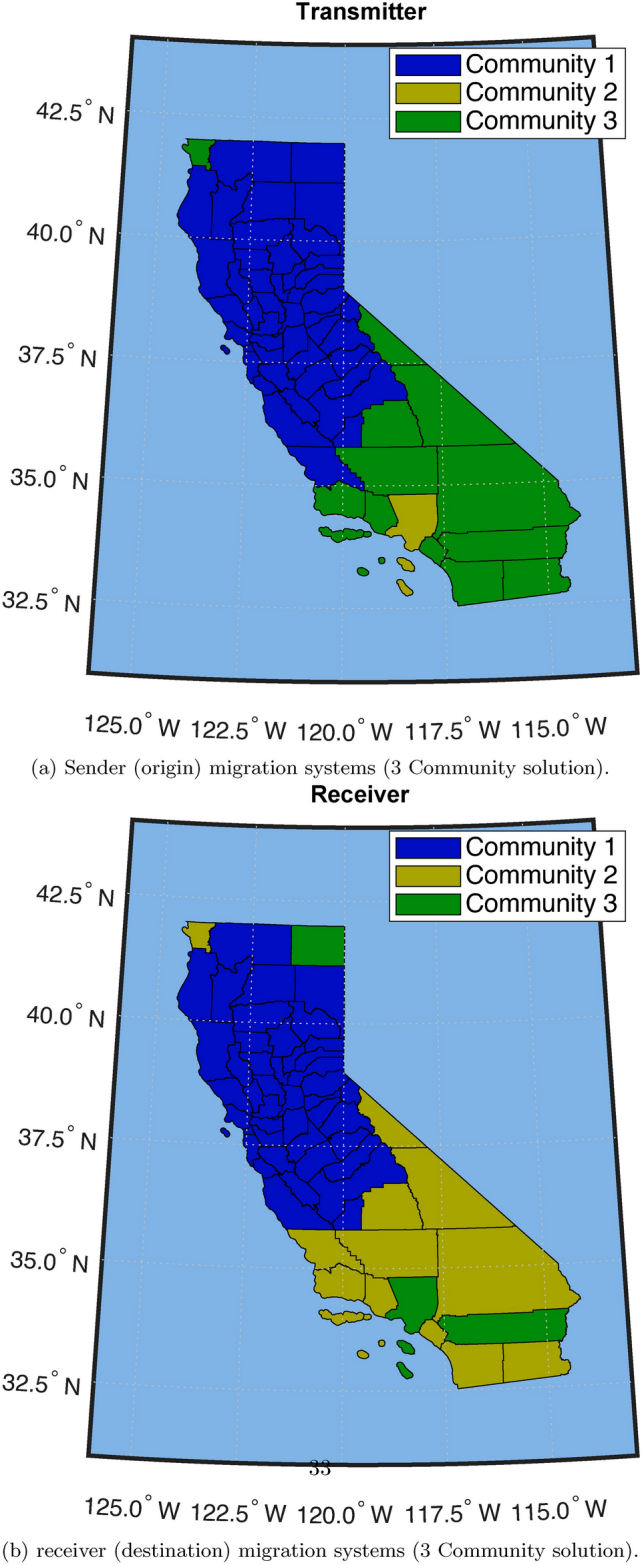
These two plots show a fully classified California for origin/destination migration systems under a three-system model. Here in this model, we see a clear Northern and Southern California division.

Given these two systems, we might be interested in classifying the whole state as three Community systems using our method combined with a clustering algorithm (in this case, k-nearest neighbors;^65^). In Fig. 4, we can see the Northern California versus Southern California split, with Los Angeles being Southern California’s core origin/destination system. A noticeable implication is that Santa Barbara County is classified as part of the Southern California system. There is active research on where people divide Southern and Northern California cognitively^66^ with Santa Barbara typically being the dividing line of Northern and Southern California in regional identification tasks^66,67^. This places further evidence of the importance of the Santa Barbara divide and whether it should be placed in Northern or Southern California.

### IRS data—Hurricane Katrina, 2005

**Fig. 5:**
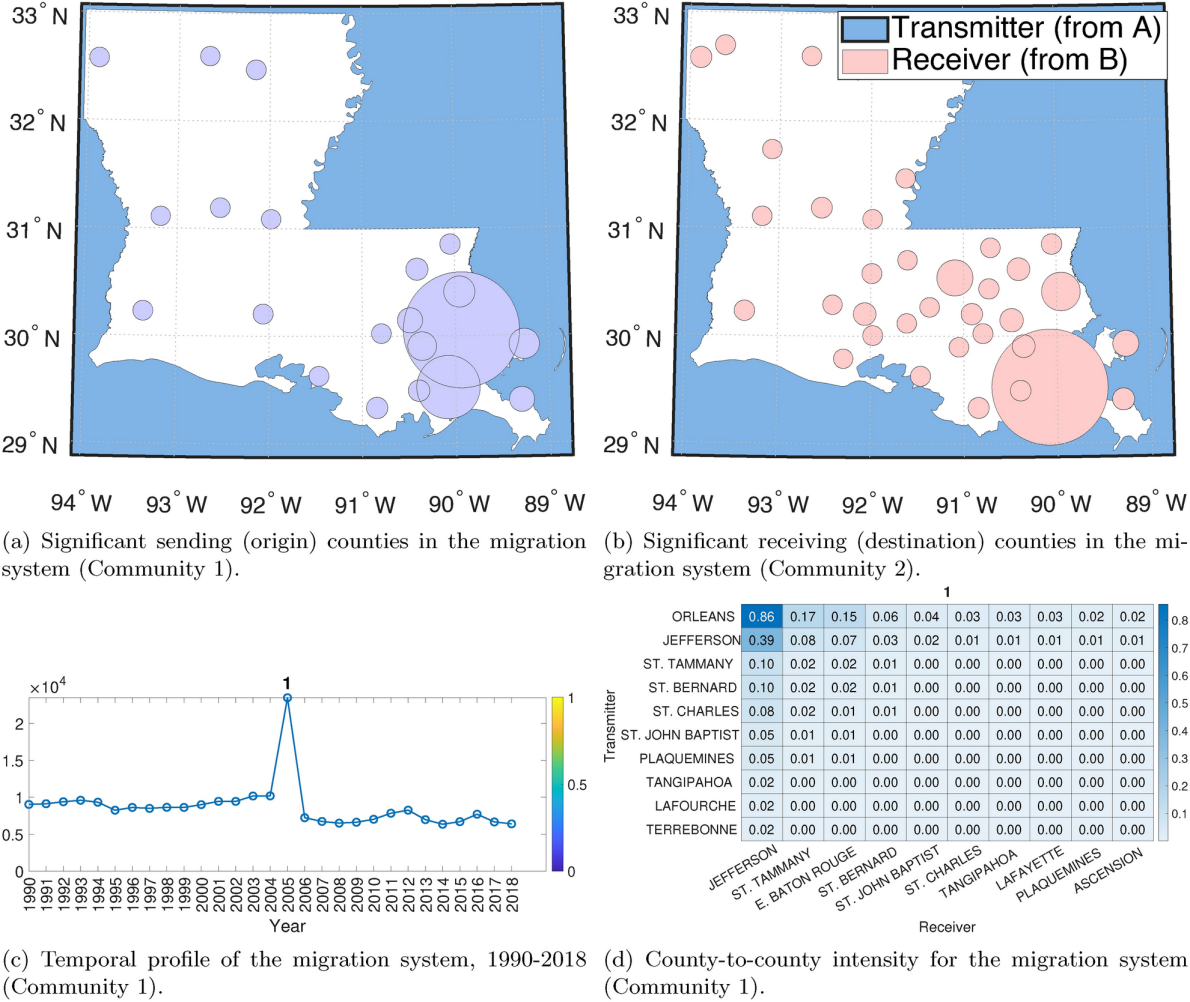
Spatial and temporal plots of the first migration system (Community 1) obtained using the proposed ST tensor method applied to Louisiana. This system illustrates a large temporal shift in 2005 when Hurricane Katrina made landfall.

One particularly compelling aspect of the ST tensor co-clustering method is the ability to detect the activity intensity changes in the migration system in response to external shocks. In 2005, Hurricane Katrina’s effect on the City of New Orleans provided an extreme example of how severe weather events can change the demographics of a major city^68,69^. In this section, we look at the migration system between New Orleans Parish and all other counties within Louisiana, as well as a node representing all the combined counties outside the state. Pre-disaster is defined as before 2004, and recovery as 2007–2009^69^. In Fig. 5 and Appendix Fig. E.12, we can distinctly see the effects of Hurricane Katrina on the migration systems in Louisiana and the City of New Orleans specifically.

#### Migration system activated by Hurricane Katrina

In Fig. 5 (Community 1), we can see the migration out of New Orleans Parish. In this case, the system engaged after the natural disaster (Community 1) differs from the one engaged for recovery (Community 2, though they share some counties in common). The key finding here is that we can see exactly which migration systems are activated for the displacement event (Hurricane Katrina) and which are activated for return migration (recovery). Further, we can see that while there is overlap in the counties, it is not the same Communities involved in the recovery—suggesting that some of the recovery is driven by new migration to the area. This can be seen in Fig. 5.

#### Migration recovery system from Hurricane Katrina

In Appendix Fig. E.12 (Community 2), we can see the recovery of the migration systems, which has been defined as 2009^68^. This method allows us to see this change in migration system activation due to exogenous shock and to see how people activate different migration systems depending on a particular event like Hurricane Katrina.

#### Comparison with alternative methods: case of Hurricane Katrina

**Fig. 6:**
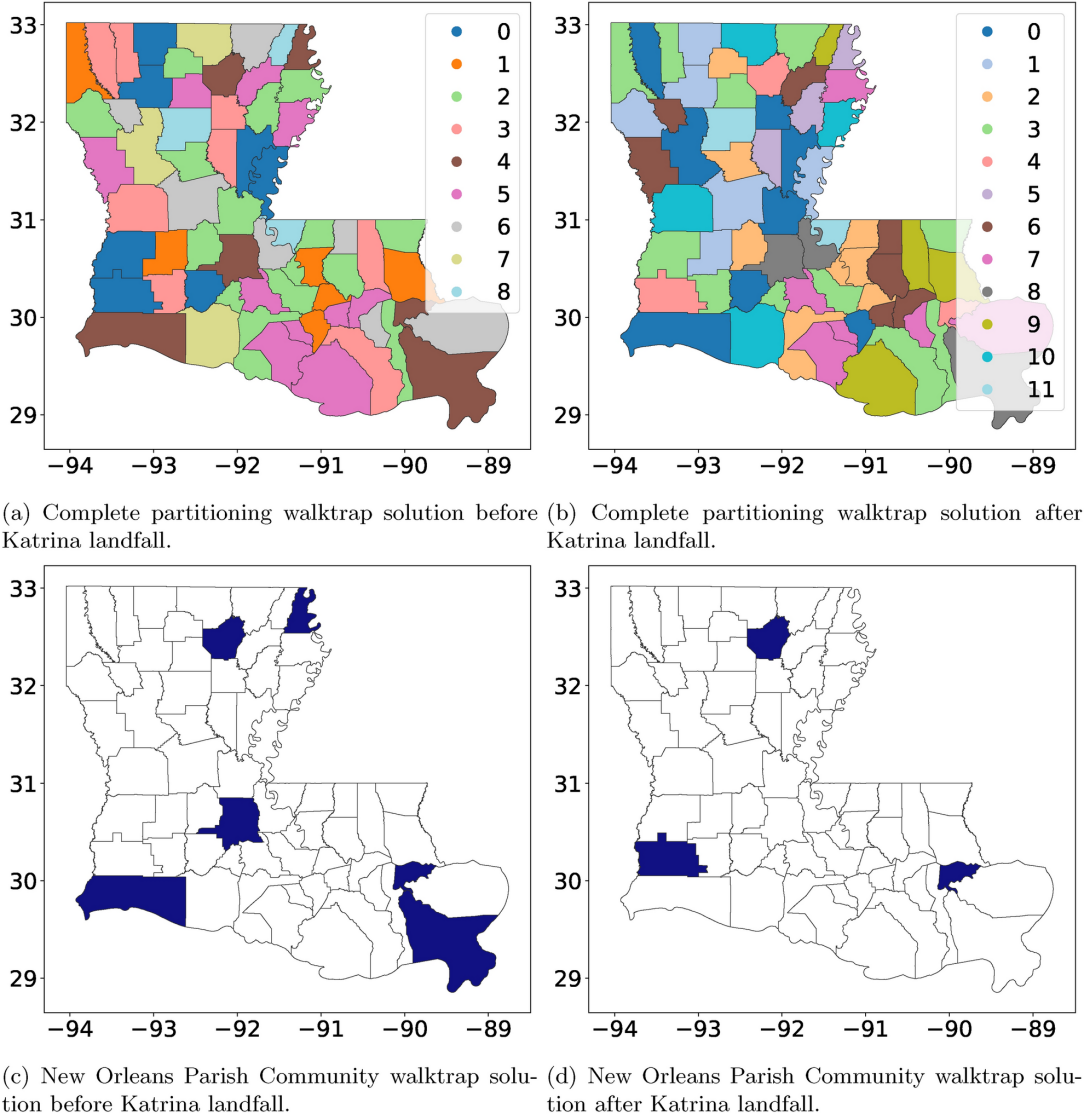
(**a**) and (**b**) is the full partitioning via the walktrap Community detection algorithm for all of Louisiana. (**c**) and (**d**) are the New Orleans Parish Community, established pre- and post-Hurricane Katrina in 2005, based on the walktrap Community detection solution.

**Fig. 7:**
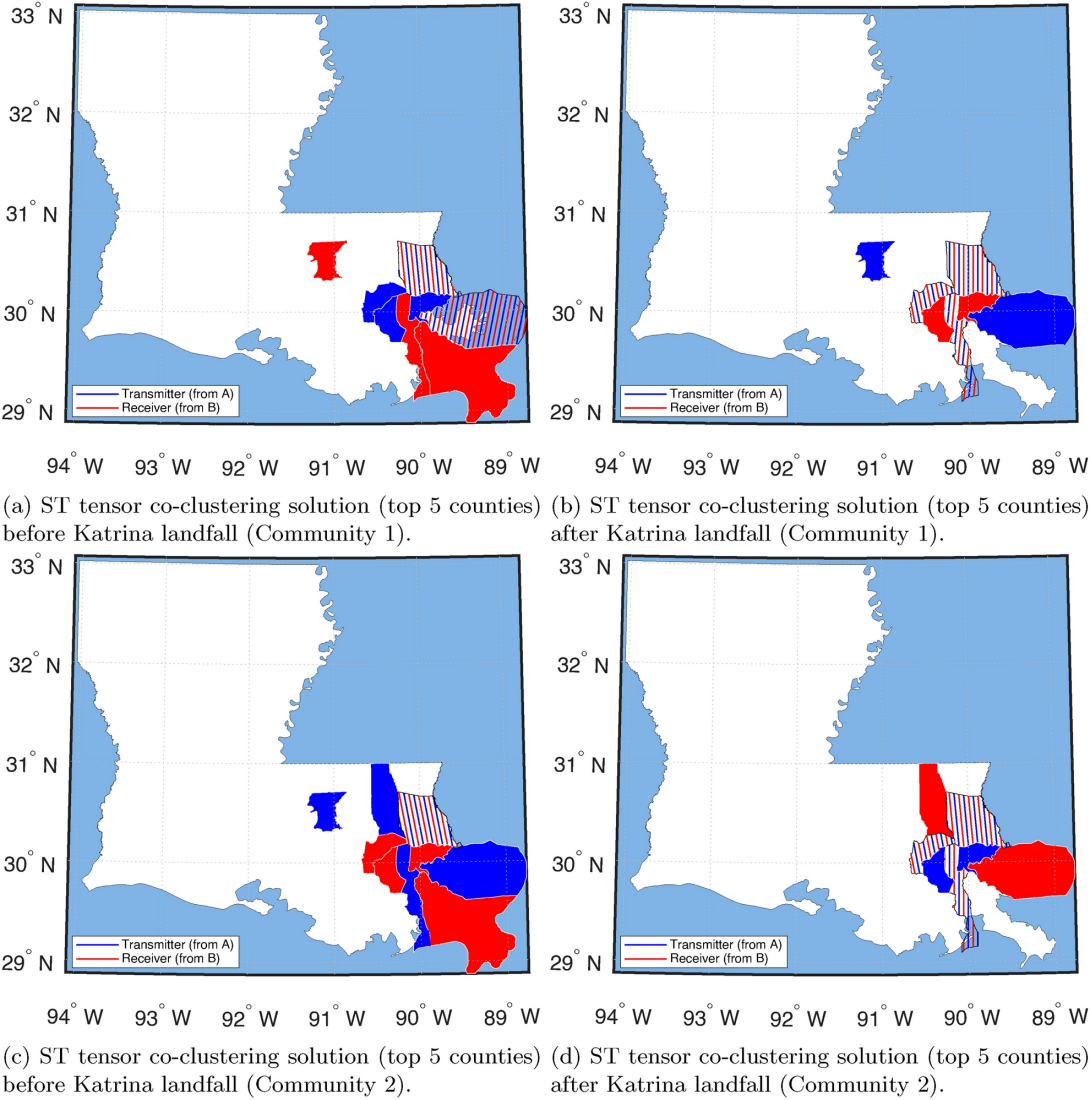
The New Orleans Community output by the ST tensor co-clustering method. Communities are displayed with their top 5 transmitters and top 5 receivers. New Orleans Parish appears in the top 5 transmitters/receivers in Communities 1 and 2, which are also the top 2 significant Communities for this system.

A typical strategy for dynamic graph clustering is to apply a classic ‘static’ Community detection technique designed for graphs without the temporal dimension (such as the walktrap method^31^) to the graphs collected at different time-points separately and look at how the resulting system changes. Here, we use a walktrap algorithm on the Louisiana domestic migration network split into pre- and post-Hurricane Katrina. We then compare the ST co-clustering method with the walktrap method (see details in Materials and Methods). The walktrap method is a random walk-based Community detection algorithm. It provides hard, nonoverlapping clustering of the counties based on their migration patterns. However, the method does not tell which migration system exhibits higher activity levels as revealed in our method; see (Fig. 6). To better compare with the ST co-clustering method, we observe the cluster (i.e., a migration system) containing New Orleans, the prominent city and the one hit heavily by Katrina (Fig. 6). In Fig. 7, we look at the top 5 origin (sender) and destination (receiver) counties found by the ST tensor co-clustering method. Note that walktrap does not offer such sender/receiver information. Next, we observe differences in cluster patterns, with Communities 1 and 2 producing the closest to the walktrap solution (see Fig. 6). According to the walktrap method, the primary system in New Orleans shrinks by half between pre- and post-Hurricane Katrina, representing the changes in the system. However, in the ST co-clustering method, we see the local cluster around New Orleans with only East Baton Rouge Parish being in the system, suggesting that the evacuation was much closer to the disaster center than the walktrap method would suggest.

### International migration data—global migration systems

Identifying migration systems in the international context remains an open problem in the field. Several works have posited that there should exist international migration systems^1–4^ and have provided a set of “general principles” of such systems rather than analytic approaches^8^. Recent work by Abel et al.^8^ has applied static Community detection methods to the international migration data provided by Azose and Raftery^48^ and updated by Abel et al.^8^ to demonstrate the change in migration systems over time. In^8^, the migration systems were found year-by-year by repeatedly applying the Community detection method to each year’s data.

Here, we apply the ST tensor co-clustering method to the same data. This clustering results in essential differences in output and understanding of the migration systems. First, ST tensor co-clustering produces a set of migration systems that exist over the entire period (though the intensity of their importance varies over time) and thus represent a cleaner set of migration systems than those in^8^. By fitting Community detection methods year by year, Abel et al.^8^ cannot guarantee the persistence of a given system. This means that the method does not ensure the discovery of clusters (networks) of countries with similar spatial interactions and varying activity intensity over time. Thus, the ST co-clustering model produces a better representation of the migration system, especially under what Kritz and Zlotnik^3^ described as “network[s] consisting of sets of the concept of dynamic stability” (see also^3^).

**Fig. 8:**
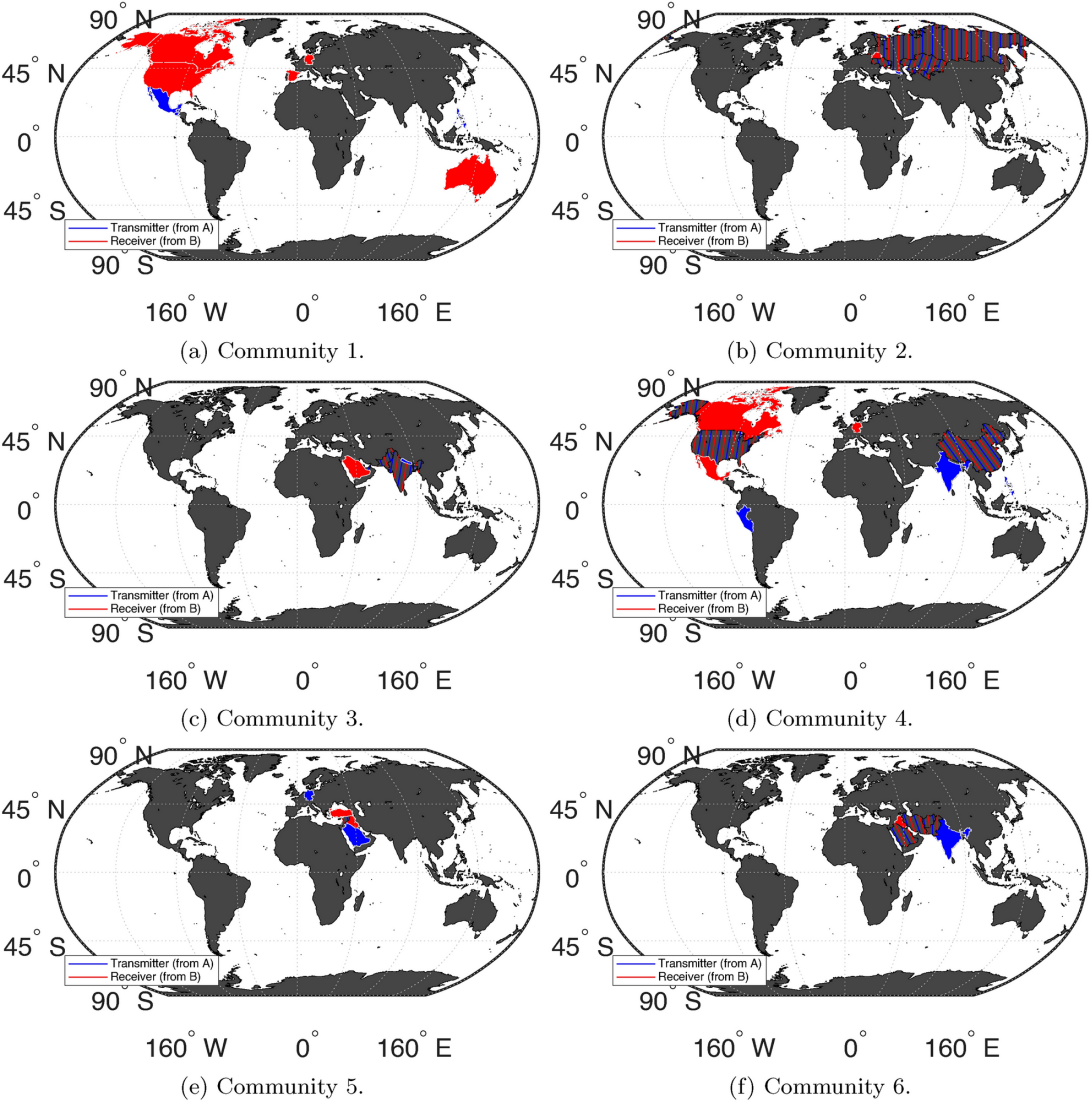
Top six migration systems for world migration for top 10 origin and destination countries from the ST tensor co-clustering method. Red is a significant destination country, and blue is a significant country of origin in the migration system. Striped indicates the country is a significant origin and destination country in the migration system.

We explore six major Communities consisting of the top 10 origin and destination locations from 1990 to 2015 (Fig. 8 and Appendix Fig. E.13). This set was chosen based on the least square fit of the data (see the Online Appendix for details). The first migration system (Appendix Fig. E.13; Community 1) is dominated by the relationship between Mexico (origin) and the United States (destination) and is characterized by countries sending migrants to the United States. The second migration system (Appendix Fig. E.13; Community 2) is characterized by Eastern European migration, with Russia dominating both the origin and destination of the system. The third migration system (Appendix Fig. E.13; Community 3) is characterized by India, Bangladesh, and other Southern and South Eastern Asian countries. The fourth migration system (Appendix Fig. E.13; Community 4) is characterized by the United States, China, and India as the largest origin countries with destination countries Mexico, South America, Asia, and Western Europe, and Russia as the primary set. The fifth migration system (Appendix Fig. E.13; Community 5) is dominated by the Middle East, with Syria being the largest origin country. Last, the sixth migration system (Appendix Fig. E.13; Community 6) is also in the Middle East and comprises Iran, Pakistan, and Afghanistan.

We have visualized these Communities with world maps in Fig. 8. In Appendix Fig. E.14, we provide examples of the temporal profile and the spatial interaction matrix of the top 10 countries in migration systems 1–6. The matrix is produced by instantiating $\widetilde{\textbf{a}}_f(i)\widetilde{\textbf{b}}_f(j)$ as its (*i*, *j*)th element, where $\widetilde{\textbf{a}}_f \in \mathbb{R}^{10}$ and $\widetilde{\textbf{b}}_f\in \mathbb {R}^{10}$ are the sub-vectors of ${\textbf{a}}_f$ and ${\textbf{b}}_f$ holding the top-10 strongest elements, respectively. The temporal profile allows us to see major events, such as the end of the Syrian occupation of Lebanon in 2005 (Appendix Fig. E.14; Community 4). Altogether, the results are similar to those of^8^ with major migration systems centering around the United States with distinct European and Eastern European systems and Middle Eastern and Southern Asia systems. We also find evidence of change in the importance of the United States-dominated migration system from migration system 1 (Community 1; Appendix Fig. E.14) to migration system 3 (Community 3; Appendix Fig. E.14) in 2005–2010, which is similar to what was described in^8^. However, we can see that this change started at the beginning of the period (Community 3; Appendix Fig. E.14), with the peak change occurring in 2005–2010. Further, because we have a stable set of countries in our system, we can see precisely how and when one system versus another becomes dominant from the temporal profile change of two US-dominated systems.

### Comparison with prior works: world migration systems

**Fig. 9:**
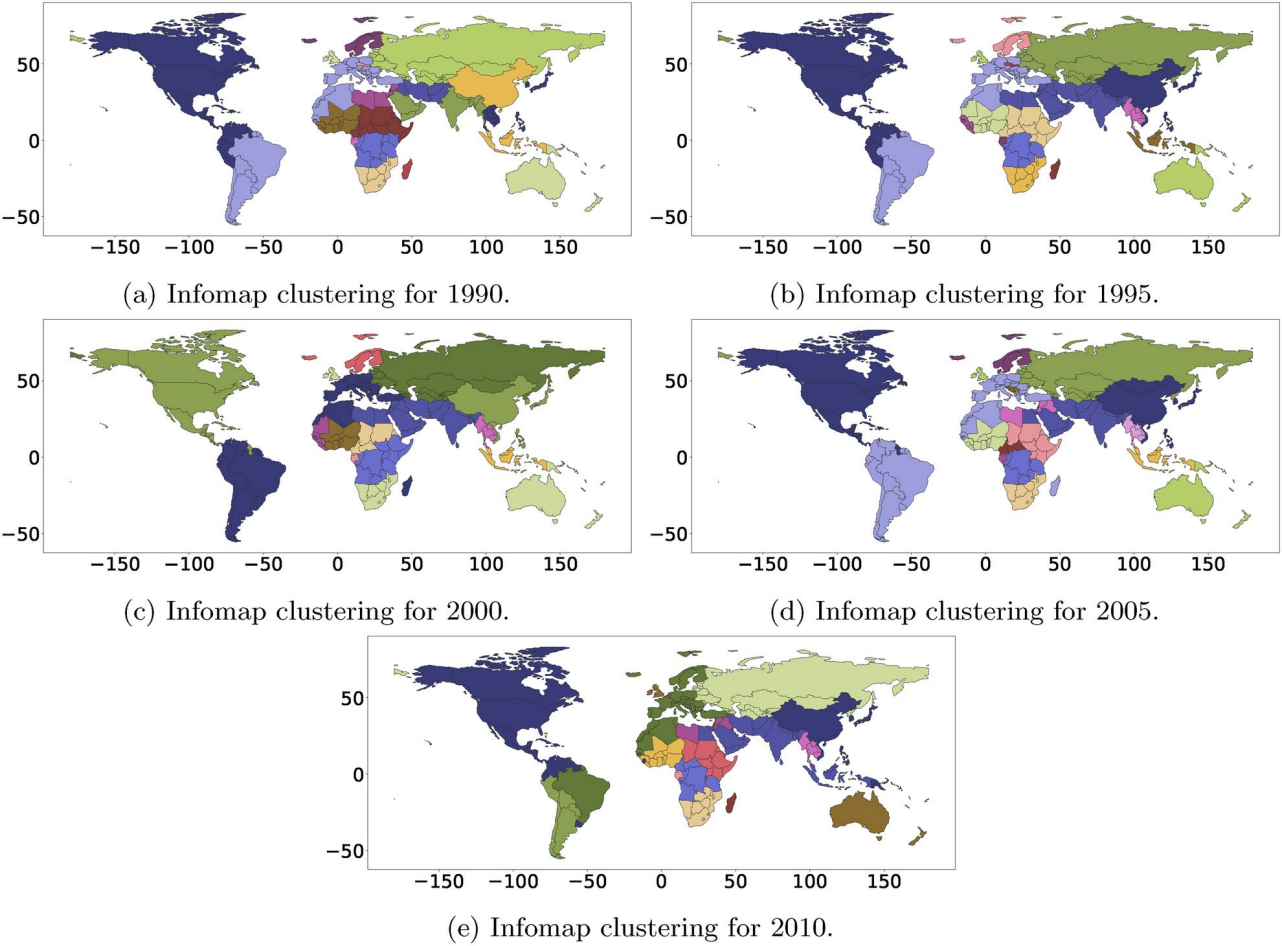
Migration systems discovered by method infoMap^14^ for international migration for the period 1990–2015 for comparison with ST tensor co-clustering algorithm.

**Fig. 10:**
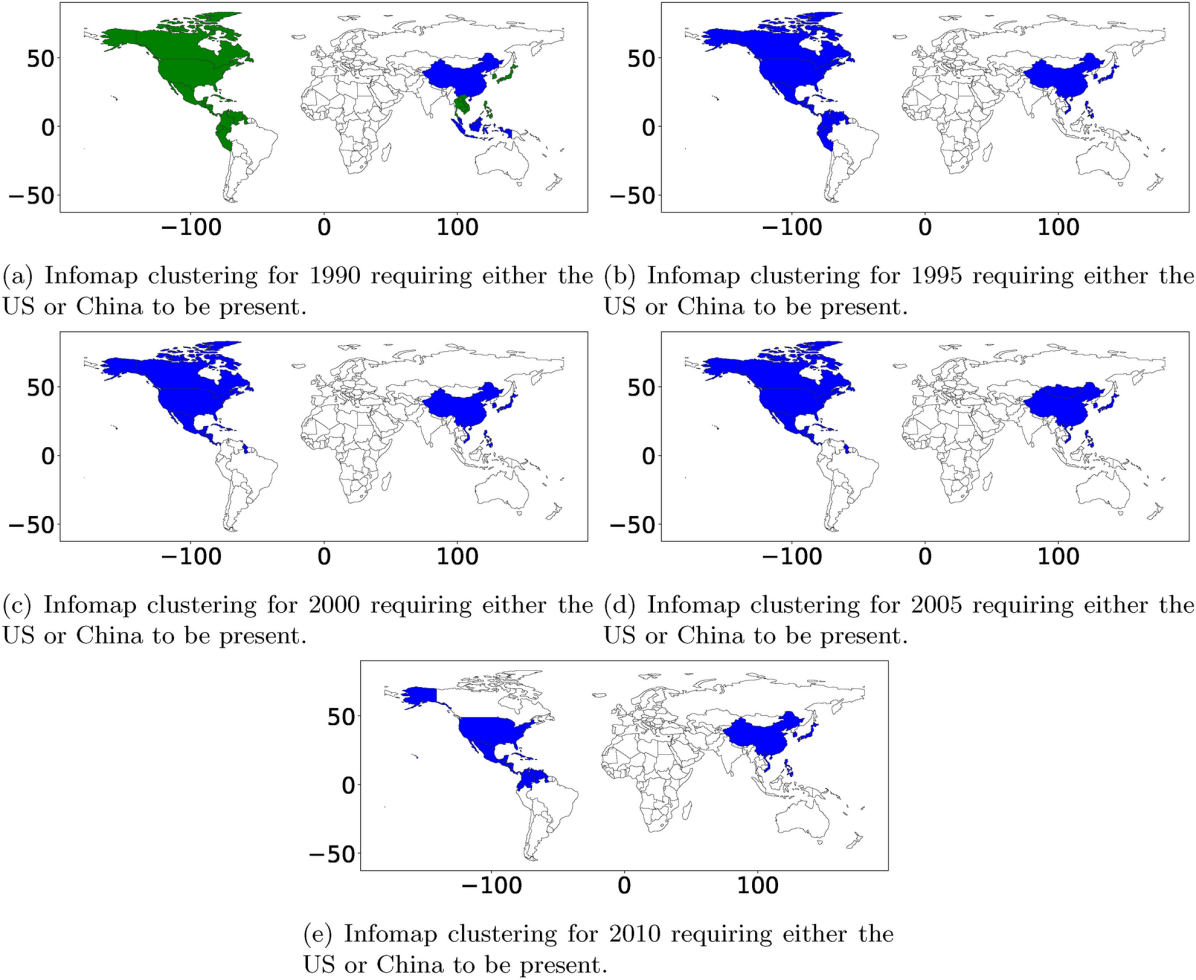
A subset of Communities selected by requiring the US or China to be in the cluster from Fig. E.9. Green and Blue represent distinct clusters. In 1990, the US and China were in distinct clusters, and from 1995 to 2010, they were contained in the same cluster by the infomap algorithm.

The work in Abel et al.^8^ employed infoMap^14^ to demonstrate the efficacy of finding migration systems using static network clustering techniques. Both methods generate a distinct North American cluster, European and Asian cluster; however, the technique of in^8^ does not provide distinct origin and destination clusters or highlight which Communities are more significant over a given time period. We have recreated these clusters in Appendix Fig. E.9 and isolated the Communities that include either the United States or China (Appendix Fig. E.10). Again, one distinct difference is that the classic methods provide a complete partitioning and focus on how these Communities evolve. In contrast, our method provides a quantitative measure and order of how “important” a Community is and how stable the Community is over time. Both methods detect the US–Canada–Mexico cluster, but our method also detects separately the China–US origin-destination cluster, where we can distinguish between whether the origin or destination is driving the relationship (see Appendix Fig. E.10 in comparison with Appendix Fig. 8). Altogether, the ST Co-Clustering method provides a distinct and valuable solution that differs from the current focus on change. Instead, ST Co-Clustering aims to uncover stable migration systems over time, as hypothesized in the literature (see Figs. 9, 10 demonstrating this difference). It further allows the ability to rank/prioritize these systems and distinguish between origin and destination clusters.

**Syrian occupation of Lebanon** Last, another key difference is our ability to spot major changes (e.g., the end of the Syrian occupation of Lebanon in 2005, which can be seen clearly in Appendix Fig. E.13e)—which does not appear in the work of Abel et al.^8^.

## Discussion

Unlike historical methods^70–75^, we provide a holistic framework for dealing with space and time in migration patterns for understanding migration systems. Our method also allows for improvements on Pandit’s^76^ migration system. Pandit sets out a “subsystems” and migration “typologies” framework, where subsystems represent high interconnectivity and low levels of interchange with other subsystems, and migration typologies represent clustering by the origin and/or destination of migrants. Thus, the relationship is not the interconnectedness between areas but rather the relative similarity of their flows within spatial units. This work provides a novel way to differentiate subsystems and typologies. Specifically, our method allows for measuring the change in typologies over a given spatial region (e.g., the effects of Hurricane Katrina) and a general measure of subsystems that is responsive to the temporal nature of migration systems, unlike historical methods like^76^ that rely on simple principal component analysis (PCA). In general, we can view this as the natural extension of what has been done historically in the empirical system, finding literature in the social sciences but accurately taking into account time and space in a way that is not currently done in the field. Further, it allows researchers interested in migration systems to go beyond simple changes in static snapshots of migration systems to holistic models of temporal stability and large-scale shifts in migration typologies.

**Limitations:** This method definitionally pulls out the spatial/temporal pattern and does not quantify “change” *per se* in the system. For example, if you want new systems over a range of years, you need to discretize the use of this method so that it would act more like the infomap or walktrap solutions we discuss as comparisons; however, even in that context, this method performs quite well (see our IRS Data—Hurricane Katrina: Louisiana comparison with walktrap algorithm or International Migration Data—Global Migration Systems comparison with the infomap algorithm). Overall, this method performs very well in terms of the intuitive definition of the migration system discussed in the literature. This method generally requires complete network data with multiple relations (time being the one focused on in this article). Future work will look to applying this method to sampled network data, but it is an ongoing research problem.

In summary, our findings have five crucial implications: (i) empirically derived migration systems can be established as stable over time and space (domestic and international migration); (ii) we can correctly derive expected migration systems across the US (e.g., Northern and Southern California) and in international contexts; (iii) we can detect exogenous shocks to the migration system (e.g., Hurricane Katrina or the end of Syrian occupation of Lebanon); (iv) we can establish changes in migration systems over time (e.g. Syrian refugee crisis); and (v) we provide a novel approach to dynamic community detection that focuses on stable clusters over time rather than change in clusters over time. Beyond the purely descriptive, these quantitative data-driven methods have the potential to improve population forecasting^77^, as Andris et al.^78^ demonstrated the usefulness of migration clusters in predicting future migration flows or potentially could be integrated in formal demographic models such as those used in Massey et al.^79^. Further, these methods could be employed to build complex statistics for exponential random graph models, which have been applied successfully to migration networks (see, for example,^60^). Tensor co-clustering is powerful for finding spatio-temporal clusters in networks like migration systems, and it also has strong potential for broader impact in the social sciences beyond migration systems in areas such as partisan politics or alliance formation.

Further, this method has the potential to change how we think about community detection in the larger social network and network science literature—allowing us to consider not just two dimensions (a typical social network) but k-dimensions for many multiplex^80^ relations (e.g., Friendship, Acquaintanceship, Kinship, Job Leads, homelessness over a single set of actors), not just time. This larger scientific endeavor under multiplexity is of general interest to social networks and the social science community.

## Methods

### Datasets

**IRS data** The IRS migration data are created in the following manner: (1) taxpayer identification numbers (TINs) are used to match tax returns in consecutive years; (2) matched tax returns where migrant returns are defined as those that do not match the state or county of residence in consecutive years; (3) total counts of tax returns and tax exemptions—effectively households and individuals respectively—and the total adjusted gross income or AGI contained in the migrant and non-migrant returns are aggregated up to the state and county levels^50–52^. Four major limitations^50^ of this data are discussed in the, which include: (1) by definition, these data do not include those who do not file a tax return. This group is disproportionately elderly and/or poor^50^. (2) The data is limited to aggregate counts of the county (or state) data on three variables: (i) total counts of migrant and non-migrant returns (i.e., households), (ii) exemptions (i.e., individuals), and (iii) AGI. (3) There is a methodological change between the 1990–2011 data and the 2012–2018 data^81^. In the 2011–2012 tax year, the data preparation shifted from the US Census Bureau to the IRS, which expanded the window for returns included in the estimates to go through December rather than September. (4) The last criticism is that for privacy reasons, the county-to-county migration flows involving less than ten households are obscured^81^. This was increased to 20 in the 2011–2018 periods^81^.

Dewaard et al.^81^ describe the limitations of using the combination of the 1990–2018 period due to processing standards change from US Census Bureau to IRS workers. Nonetheless, because the ST tensor co-clustering method works in a low-rank approximation manner analogous to the principal component analysis (PCA) for matrix data, such measurement error-induced noise is not expected to cause visible issues. Further, to evaluate the robustness of this assumption, we have done a series of tests (available in the Online Appendix), and no major red flags have appeared. So while^81^ does caution against this practice, we find our procedure robust to the issues discussed.

Alternative migration data in the US lacks temporal and spatial resolution for such an analysis. For example, there exists one-year migration estimates from the 2000 US Census long form (1 year of data) and five-year interval estimates from the American Community Survey (ACS) from 2010 to 2019 (i.e. 2–3 years of data). However, neither of these estimates provides temporal resolution of the IRS data. Many county estimates are suspect because the ACS surveys do not have a large enough sample in any given year to make estimates below the state level.

**International migration data** Estimation of international migration is a complex and important area of research. Recently, Abel and Cohen^82^ updated the migration estimates from Azose and Raftery^48^, which cover 200 countries every five years from 1990 to 2015. This is the same data used in Abel et al.^8^ and the estimates we use in this article. The statistical method used to develop these estimates is built on the work by Azose and Raftery^48^, which builds on the work by Abel^83,84^.

This data is developed by first gathering data on country-level migration stocks, desegregated by country of birth based on administrative and United Nations records. Next, the researchers employ demographic balancing equations to harmonize the data between two periods. The idea is that any change in the migration stock must align with the component changes in fertility, mortality, and migration in a given country. These models use the fertility and mortality information from the United Nations World Populations Prospects to estimate the country-to-country migration flow data at five-year intervals. It is worth noting that Abel’s^83^ original construction more closely follows report data, and Azose and Raftery’s^48^ employ a Bayesian model to produce the final estimates. Abel et al.^8^ describe the following major limitations to these migration data in that statistical models do not reconcile migration reports of sending and receiving countries, which are often not in agreement. However, these harmonized and estimated migration data are generally considered the best migration data under current standards^82^.

### Spatial-temporal co-clustering model

We consider three-way data $\mathcal{X}\in \mathbb {R}^{I\times J\times K}$, where $\mathcal{X}(i,j,k)$ represents the number of individuals who moved from location *i* to location *j* in year *k*. We expect that migration occurs organically in systems largely driven by sociological, economic, and demographic factors, as well as major local events such as a natural disaster. The migration patterns are grouped through the “origin locations” (i.e., locations where people move from) and “destination locations” (i.e., where people move to) and the temporal pattern of this movement. Such a data set can be represented as in a multi-way tensor co-clustering framework^47^. Specifically, we model $\mathcal{X}$ (the origin by destination by time array) as the following decomposition:

1 $$\begin{aligned} \mathcal{X} \approx&\sum _{f=1}^F {\textbf{a}}_f\circ {\textbf{b}}_f \circ {\textbf{c}}_f, \end{aligned}$$

where $\circ$ denotes the outer product, i.e.,

$$\begin{aligned} \left[{\textbf{a}}_f\circ {\textbf{b}}_f\right]_{i,j} ={\textbf{a}}_f(i){\textbf{b}}_f(j),~ [ {\textbf{a}}_f\circ {\textbf{b}}_f \circ {\textbf{c}}_f]_{i,j,k} ={\textbf{a}}_f(i){\textbf{b}}_f(j){\textbf{c}}_f(k). \end{aligned}$$

Here, ${\textbf{a}}_f\circ {\textbf{b}}_f\circ {\textbf{c}}_f$ represents the *f*th co-cluster – a migration system over time in our context. The vector ${\textbf{a}}_f$ indicates the membership (or the degree of association) of the *I* origin locations with co-cluster *f*. For example, ${\textbf{a}}_f(i)=0$ means that county *i* is not in the *f*th migration system. The ${\textbf{b}}_f$ vector is defined similarly for the destination locations. Note that ${\textbf{a}}_f\circ {\textbf{b}}_f={\textbf{a}}_f{\textbf{b}}_f^T$ is a rank-one matrix and defines a bipartite clique (i.e., fully connected bipartite sub-network) over the origin-destination network. The vector ${\textbf{c}}_f$ scales the clique over time, which can be regarded as the clique’s temporal signature. Intuitively, ${\textbf{c}}_f(k)$ being a large value means that the *f*th migration system has intense migration activities at the *k*th year.

The model in (1) is the so-called canonical polyadic decomposition (CPD) of third-order tensors if *F* is the smallest integer that makes (1) hold exactly. In such cases, *F* is referred to as the tensor rank. Under our hypothesis, finding the CPD expression of the migration data can reveal major migration co-clusters and their activity levels over time.

The key advantage of CP decomposition is that its rank-one components are unique and can thus be better interpreted. This is to be contrasted with bilinear (matrix) factor analysis methods, which do not produce unique rank-one components. Taking SVD, for example, and absorbing the singular values into the left and right matrix factors, we can obtain another *equivalent* decomposition of the given low-rank matrix. The reason that SVD itself is unique is that we insist on the orthogonality of the singular vectors. However, the ‘true’ underlying components we seek in applications are rarely orthogonal; thus, SVD fails to unravel them.

Owing to the inherent uniqueness of CP decomposition, we cannot enforce its components to be orthogonal, as the true generating latent factor matrices are not orthogonal. The result is that the variance explained by the sum of CP components is not the sum explained by the individual components, so we cannot talk about the variance explained by a single component in isolation, as in SVD. However, we can extract a set of *F* principal CP components, which best explain the given data. Because they are unique, there is no ambiguity in visualizing them, as is the case with the matrix.

To be more precise, the co-clustering algorithm tackles the following optimization problem:

2 $$\begin{aligned} \mathop {\textrm{minimize}}\limits _{\textbf{A}, \textbf{B}, \textbf{C}}&\quad \left\Vert \mathcal{W} \circledast \left( \mathcal {X} - \sum _{f=1}^F {\textbf{a}}_f\circ {\textbf{b}}_f\circ {\textbf{c}}_f \right) \right\Vert _F^2, \nonumber \\ \text {subject to}\quad&~\textbf{A}\ge \textbf{0}, \textbf{B}\ge \textbf{0}, \textbf{C} \ge \textbf{0}, \end{aligned}$$

where $\textbf{A}=[{\textbf{a}}_1,\ldots ,{\textbf{a}}_F]$, $\textbf{B}=[{\textbf{b}}_1,\ldots ,{\textbf{b}}_F]$, $\textbf{C}=[{\textbf{c}}_1,\ldots ,{\textbf{c}}_F]$, $\mathcal{W}\in \mathbb {R}^{I\times I\times K}$ is a weight tensor such that

$$\begin{aligned} \mathcal{W}(i,i,k)=0,~\forall i,~\forall k,~~~\mathcal{W}(i,j,k)=1,~\forall k,~\forall i \ne j, \end{aligned}$$

and $\circledast$ denotes the Hadamard product. The nonnegativity constraints imposed on $\textbf{A}$, $\textbf{B}$ and $\textbf{C}$ reflect their physical interpretations. The weighting discards the diagonal entries in each slab of the data tensor since entries like $\mathcal{X}(i,i,k)$ always dominate in magnitude. Still, they represent static residents in county *i* and do not encode movements.

**Algorithm, software, and hyperparameter selection** The formulation in (2) entails a special tensor completion problem. Many off-the-shelf algorithms have been designed to handle this problem and its variants; see^47,85,86^. In this work, we employ the well-optimized and freely available Tensorlab software toolbox^87^ to solve the formulated problem in (2). Tensorlab is a Matlab toolbox that is widely used in the signal and data analytics Community. The software has a suite of flexible functions that can deal with plain-vanilla tensor decomposition and tensor decomposition with multiple constraints, e.g., nonnegativity, sparsity, and smoothness. The software can also easily handle missing values. In a nutshell, tensorlab treats a wide range of tensor decomposition problems as a nonlinear least squares problem and recasts these problems into a form that can be dealt with using a Gauss-Newton (GN) nonlinear programming framework. The subproblems in the GN framework are handled using conjugate gradient, which can effectively exploit the multilinear structure of tensor problems to develop lightweight updates. A tutorial of tensorlab’s basic framework and updating rules can be found in Ref^85^. Users unfamiliar with tensors and nonlinear programming may also use tensorlab as a black box.

The proposed method selects only one hyperparameter, the model’s tensor rank, corresponding to the number of migration systems. The tensor rank is analogous to the number of principal components in the matrix principal component analysis (PCA) case. For real-life data, due to noise and modeling error, the data tensors tend to have high (or full) rank. However, the tensor’s “useful signal part” is believed to have a low rank due to the high correlations across different modes. Unlike matrix PCA, incrementally extracting *F* components from the tensor one by one does not ensure that one will extract the *F* best (most significant) rank-one components from the data—due to the lack of orthogonality of the latent factors. Furthermore, extracting the principal CP component is NP-hard in general; see^46^ and references therein. Nevertheless, we do have good software tools such as tensorlab that work very well in practice, and when the latent factors $\textbf{A}$, $\textbf{B}$ and $\textbf{C}$ are nonnegative and sparse. Even incremental extraction often produces the most prominent *F* components, as observed in Ref^47^. In our case, the latent factors are indeed nonnegative and sparse, and thus, we have good reason to believe that the $F=6$ migration systems extracted from both datasets are the most prominent ones. In the Online Appendix, we present evidence supporting our choice of this single hyperparameter, i.e., setting $F=6$. It turns out that further increasing *F* does not change the first six Communities significantly, which validates our postulate.

### Related works

The co-clustering idea was first introduced in Ref^47^ for discovering Communities from email networks over time. Tensor-based co-clustering was also found useful in analytical chemistry^88^. Variations of tensor co-clustering were recently used for football team clustering, Wikipedia user clustering, and autonomous systems analysis^89^. In terms of migration data analysis, a short workshop paper presented preliminary results of using tensor models to discover the most significant migration clique spatio-temporal migration data. There, instead of using optimization-based low-rank decomposition as in our work, a Bayesian inference framework was used, where the migration counts were modeled as drawn from Poisson distributions and the factor matrices were given Gamma priors^90^. The Bayesian nature of the work in Ref^90^ may make the method heavily dependent on priors, which are not known for real-world data. Non-parametric approaches and those that use as few assumptions and parameters as possible are preferable for exploratory analysis.

### Comparison with dynamic approaches to community detection

**Comparison methods: Walktrap** To detect any changes in the migration pattern before and after Hurricane Katrina, we construct an aggregated pre-Katrina migration matrix and an aggregated post-Katrina migration matrix. More precisely, let $\textbf{W}_{\text {pre}} =\sum _{i=1990}^{2004}\textbf{W}_i-\text {diag}\left( \sum _{i=1990}^{2004}\textbf{W}_i\right)$ denote the aggregated pre-Katrina weight matrix, where $\textbf{W}_i$ denotes the weight matrix associated with the *i*-th observation period and $\text {diag}\left( \sum _{n=1990}^{2004}\textbf{W}_i\right)$ is a diagonal matrix that holds the diagonal elements of $\sum _{i=1990}^{2004}\textbf{W}_i$ on its diagonal. Note that we do not have details on migration patterns within the county; thus, the diagonal elements of $\textbf{W}_{\text {pre}}$ are set to zero. We construct the aggregated post-Katrina migration matrix ($\textbf{W}_{\text {post}} =\sum _{i=2006}^{2018}\textbf{W}_i-\text {diag}\left( \sum _{i=2006}^{2018}\textbf{W}_i\right)$) analogously. The clustering method Walk Trap proposed in Ref^31^ and applied on $\textbf{W}_{\text {pre}}$ and $\textbf{W}_{\text {post}}$ yields the Communities depicted in Fig. 6. The Community containing New Orleans is depicted in Fig. 6.

**Comparison method: InfoMAP** The InfoMAP^14^ Community detection method is an information theoretic-based Community detection technique. It receives an adjacency matrix representing a directed and weighted network (e.g., our international migration data). It produces a list of hard-clustering (i.e., full partitioning) Communities with the goal of optimally compressing information flow. InfoMAP is directly applied to the international migration data for each period. The corresponding results for the five different periods are shown in Fig. E.9. In addition, to reflect changes in community structure over time, we focus on communities, including the US and China, as these are likely the most important members. The results are depicted in Fig. E.10.

## Supplementary Information

Supplementary Information.

## Data Availability

All data and code is available through the Harvard Dataverse at https://doi.org/10.7910/DVN/EGFDU3.
