# Supplementary material for: Uncovering migration systems through spatio-temporal tensor co-clustering
Source: Sci Rep. 2024 Nov 6;14:26861. doi: 10.1038/s41598-024-78112-z (PMC11538304; doi:10.1038/s41598-024-78112-z)
Supplement: Supplementary file 1 — Supplementary Information. [file 41598_2024_78112_MOESM1_ESM.pdf]

Appendix to Uncovering migration systems through spatio-temporal tensor co-clustering

Zack W. Almquist[1], Tri Duc Nguyen[2], Mikael Sorensen[3], Xiao Fu[2], and Nicholas D. Sidiropoulos[3]

[1]Departments of Sociology & Statistics, University of Washington, Seattle, WA, 9819; Corresponding Author: zalmquist@uw.edu.
[2]Electrical Engineering and Computer Science, Oregon State University, OR, 97331
[3]Electrical and Computer Engineering, University of Virginia, VA, 22904

October 23, 2024

A Online Supplemental Appendix

The supplemental appendix reviews the algorithm/software for the analysis and methods for finding migration systems. After reviewing the necessary details, we provide background for key experiments discussed in the main document.

B Additional Figures for IRS Data - California

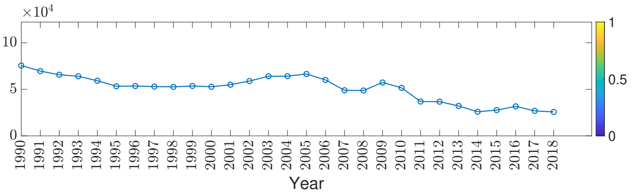

(a) Community 1.

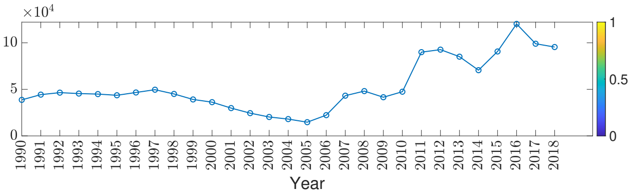

(b) Community 2.

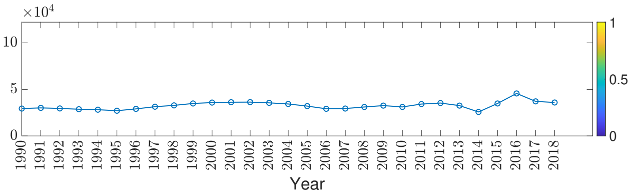

(c) Community 3.

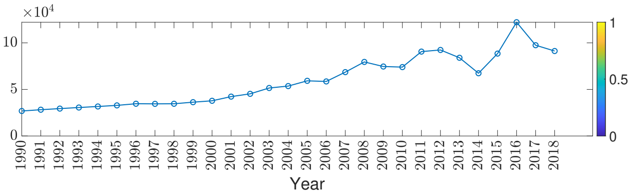

(d) Community 4.

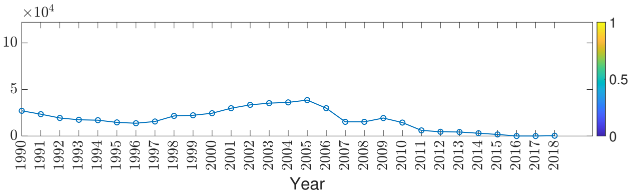

(e) Community 5.

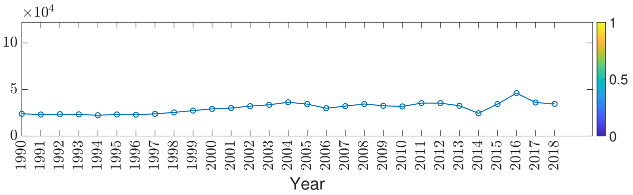

(f) Community 6.

Figure B.1: For each of the six Communities in Figure 3 there is a temporal profile for the "intensity" of the migration system over time. This temporal profile allows us to see how active this migration system is over different years. For example, we see the intensity of the Southern California migration system (Community 4) increases over time.

C Additional Figures for IRS Data - Hurricane Katrina

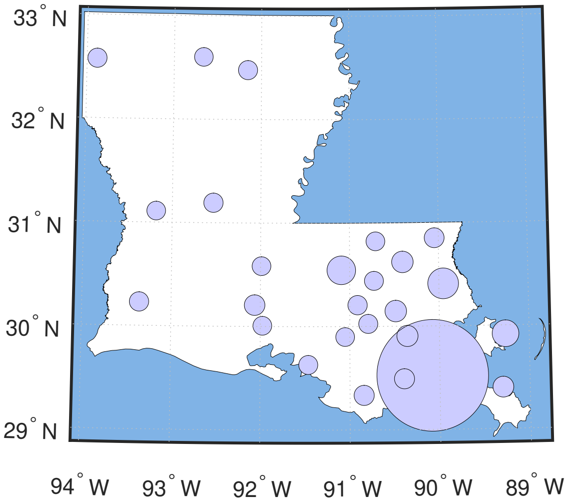

(a) Significant sending (origin) counties in the migration system (Community 2).

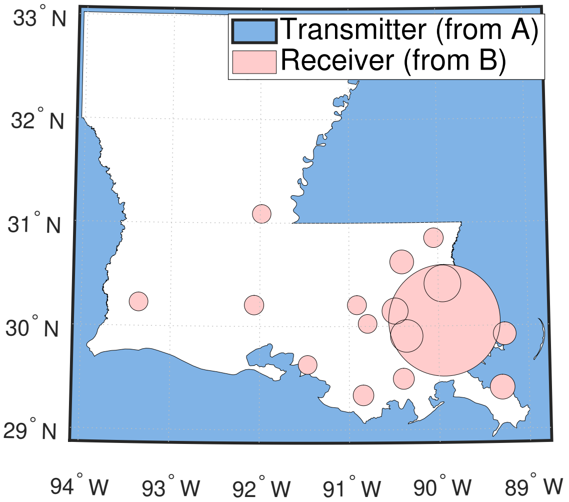

(b) Significant receiving (destination) counties in the migration system (Community 2).

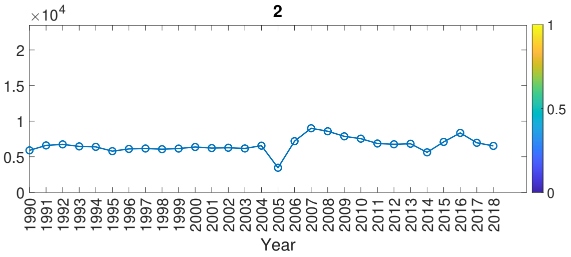

(c) Temporal profile of the migration system, 1990-2018 (Community 2).

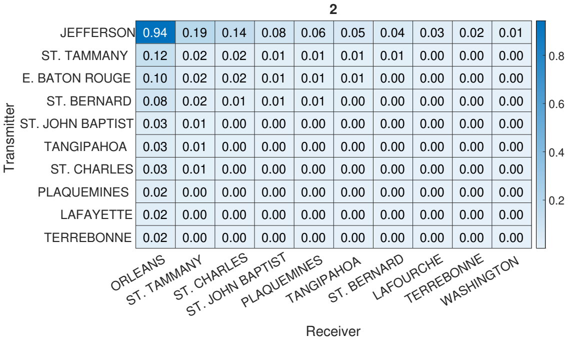

(d) County-to-county intensity for the migration system (Community 2).

Figure C.1: Spatial and temporal plots of the first migration system (Community 2) obtained using the proposed ST tensor method applied to Louisiana. These plots illustrate a large temporal shift at the recovery point from Hurricane Katrina hitting landfall in 2005.

D Additional Figures for International Migration

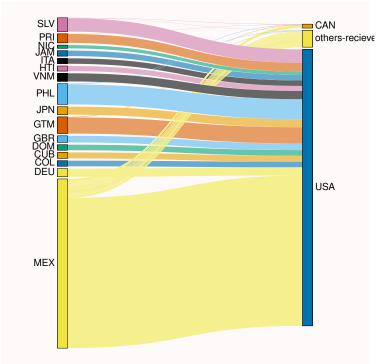

(a) Significant origin countries to destinations for Community 1.

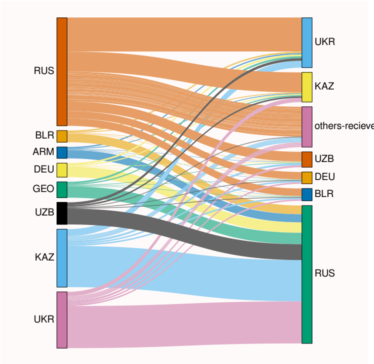

(b) Significant origin countries to destinations for Community 2.

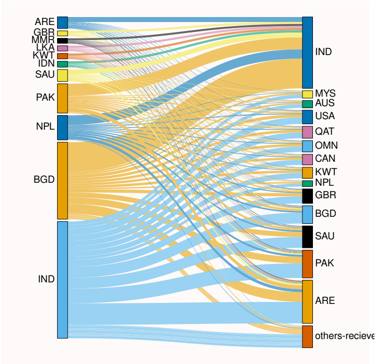

(c) Significant origin countries to destinations for Community 3.

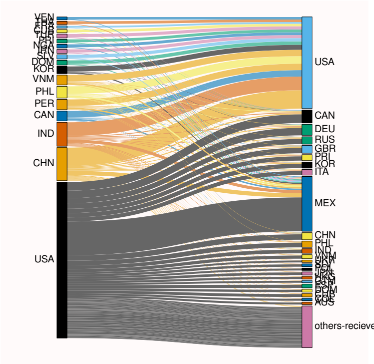

(d) Significant origin countries to destinations for Community 4.

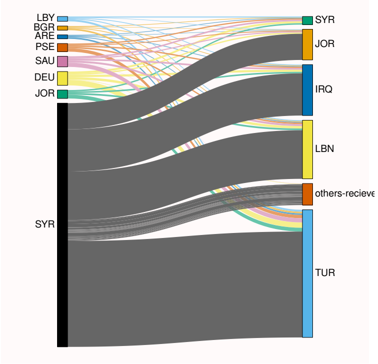

(e) Significant origin countries to destinations for Community 5.

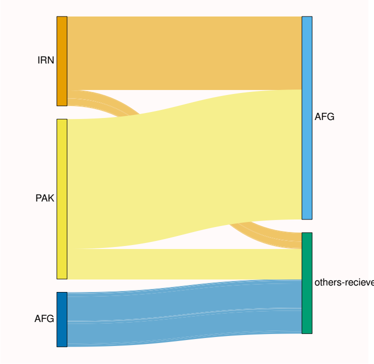

(f) Significant origin countries to destinations for Community 6

Figure D.1: Top six migration systems for international migration from 1990-2015. On the left is the main country of origin in the system, and on the right is the country of destination for the system. The size of the path between countries represents the scale of the sending or receiving migration pipeline between the two countries.

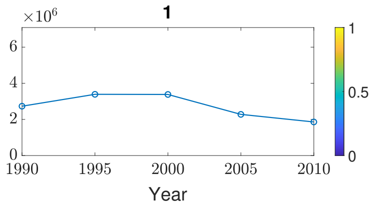

Community 1 (temporal profile)

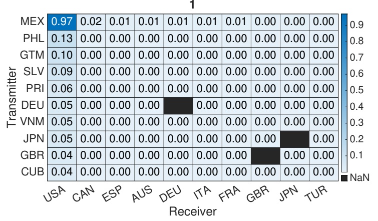

Community 1 (top 10 countries)

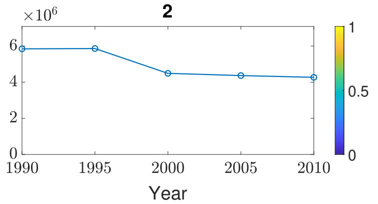

Community 2 (temporal profile)

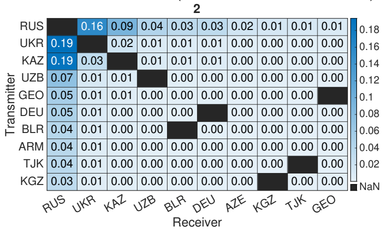

Community 2 (top 10 countries)

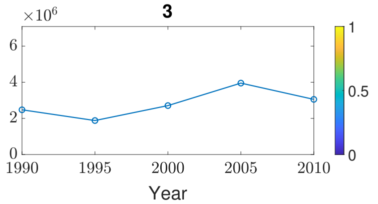

Community 3 (temporal profile)

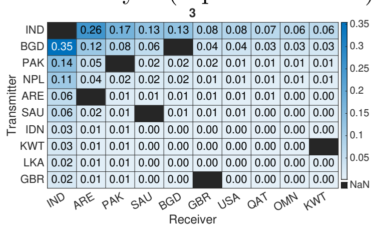

Community 3 (top 10 countries)

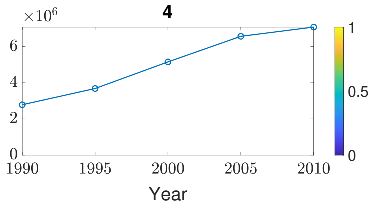

Community 4 (temporal profile)

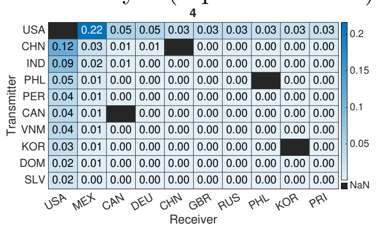

Community 4 (top 10 countries)

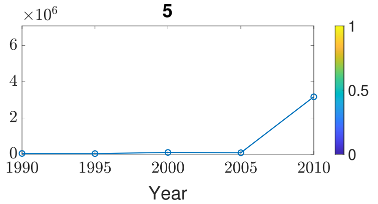

Community 5 (temporal profile)

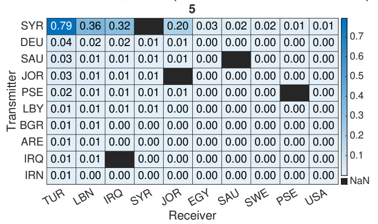

Community 5 (top 10 countries)

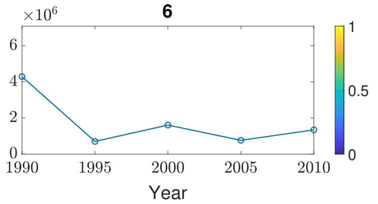

Community 6 (temporal profile)

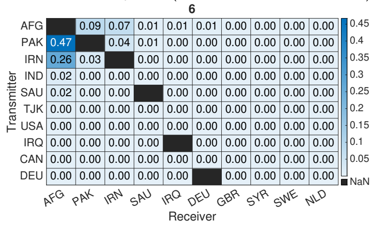

Community 6 (top 10 countries)

Figure D.2: The temporal profile (left) and top 10 countries (right) for the six migration systems estimated from the international migration data from 1990-2015. All years on the label are coded from the first year in the five-year interval, i.e., 1990-1995 is labeled 1990, and 2010-2015 is labeled as 2010.

E Model, Migration System Identification, and Visualization Methods

Data and Data Processing

Below, we review domestic data processing.

1. **IRS Data** We assume that the migration flows occur as weighted directed networks with yearly temporal aggregation. These weighted spatiotemporal networks are derived from the Internal Revenue Service (IRS) migration data matrices. Each year, the IRS data produces a data matrix, with origin (counties or states) and destinations (counties or states) determined via taxpayer records from the current and the previous year. The (i, j)th entry of the matrix represents the number of taxpayers who moved from county (or state, depending on the smallest unit that we are interested in the particular experiment) i to county (state) j from the previous tax year to the current tax year. The full data comprises migrations among 3,149 counties in the US over 29 years (1990 to 2018).

   For pre-processing, we use the 29 matrices to construct a tensor with a proper size, which is denoted as $\mathcal{X} \in \mathbb{R}^{I \times J \times K}$, where I and J ($J = I$ in our context) denote the number of origins and destinations, respectively, and K is the number of years contained in our data. Since we are interested in discovering common migration patterns, the exact number of residents staying within the same counties/states is not our primary interest. Therefore, diagonal entries in every frontal slab of the data tensor $\mathcal{X} \in \mathbb{R}^{I \times I \times K}$, i.e., $\mathcal{X}(i, i, k), 1 \leq i \leq I, 1 \leq k \leq K$, are removed and treated as missing entries. This also makes sense from the algebraic modeling point of view since diagonal entries are dominant (since the proportion of people who stay in their current counties/states overwhelms the other migration patterns), destroying the co-clustering model's low-rank structure.

Review of Model Assumptions

After obtaining the latent factors $\mathbf{A}, \mathbf{B}, \mathbf{C}$, the ith Community is identified using the rank-1 tensor

$$\mathcal{T}_i = \mathbf{a}_i \circ \mathbf{b}_i \circ \mathbf{c}_i.$$

The significance of each Community is computed based on the Frobenius norm of $\mathcal{T}_i$ after omitting all diagonal entries from $\mathcal{T}_i$—for the reason that we discussed in the previous paragraph. The spatial interactions of counties within a migration system/Community are represented by matrix $(\mathbf{a}_i \mathbf{b}_i^T)/(\|\mathbf{a}_i\|_2 \|\mathbf{b}_i\|_2)$, while the temporal association of that Community is encoded in $(\|\mathbf{a}_i\|_2 \|\mathbf{b}_i\|_2)\mathbf{c}_i$. The spatial interaction patterns and the temporal profiles then serve for our illustrations. Note that if $\mathbf{a}_i(j) > \mathbf{a}_i(q)$, this means that the involvement of the transmitter county/state j is larger than county/state (q) in the ith migration system (Community). This helps us to identify the top 5 counties/states in each migration system.

Using the above, we have a number of visualization methods as follows:

1. **Spatial Migration Maps.** The resulting Communities are (re-)constructed by the outer product $\widetilde{\mathbf{a}}_i \widetilde{\mathbf{b}}_i^T$ for $1 \leq i \leq F$ where F is the predefined tensor rank, and $\widetilde{\mathbf{a}}_i = \mathbf{a}_i / \|\mathbf{a}_i\|_2, \widetilde{\mathbf{b}}_i = \mathbf{b}_i / \|\mathbf{b}_i\|_2$.

   We color code the top-5 "transmitter" (i.e., origin) counties/states and "receiver" (i.e., destination) counties/states from the same Community in a map, see Figure 3 for example. To pick out the top-5 transmitters, the five largest elements are chosen from $\mathbf{a}_i$. Similarly, top-5 receivers are chosen from vector $\mathbf{b}_i$.

   In some instances, we also visualize all transmitters (or receivers) where node ℓth's size is proportional to $\widetilde{\mathbf{a}}_i(\ell)$ (or $\widetilde{\mathbf{b}}_i(\ell)$) — see Figure 2 as an example.

2. **Spatial Migration Sankey Diagram.** We visualize the intra-interaction within a Community using Sankey diagrams. Some examples are in Figures D.1:(a)-(f). In these figures, the width of a line connecting transmitter u on the left column and receiver v on the right column is proportional to the product $\widetilde{\mathbf{a}}_i(u)\widetilde{\mathbf{b}}_i(v)$.

3. **Temporal Profiles of Migration Systems.** A scaled factor $\mathbf{c}_i$ reflects the intensity of the ith Community varying against the time indices. In particular, as an example in Figure 2:(c), a chart shows the value $\|\mathbf{a}_i\|_2 \|\mathbf{b}_i\|_2 \mathbf{c}_i(k)$ as a function of time index k in years.

E.1 Review of the Algorithm

We employ the publicly available software `tensorlab` (1) to solve the formulated problem in the main paper, i.e.,

$$\begin{aligned} &\underset{\mathbf{A},\mathbf{B},\mathbf{C}}{\text{minimize}} \left\| \mathcal{W} \circledast \left(\mathcal{X} - \sum_{f=1}^{F} \mathbf{a}_f \circ \mathbf{b}_f \circ \mathbf{c}_f \right) \right\|_F^2, \\ &\text{subject to } \mathbf{A} \geq \mathbf{0}, \mathbf{B} \geq \mathbf{0}, \mathbf{C} \geq \mathbf{0}. \end{aligned} \tag{1}$$

The `tensorlab` toolbox is developed by the research group led by Lieven De Lathauwer at Katholieke Universiteit Leuven, Belgium (see https://www.tensorlab.net/). The software is based on Matlab and is widely used in the signal and data analytics Community. The software has a suite of flexible functions that can handle plain-vanilla tensor decomposition and tensor decomposition with multiple constraints, such as nonnegativity, sparsity, and smoothness. The software can also easily handle missing values. In a nutshell, `tensorlab` treats a wide range of tensor decomposition problems as a nonlinear least squares problem and recasts these problems into a form that can be dealt with using a Gauss-Newton (GN) nonlinear programming framework. The subproblems in the GN framework are handled using conjugate gradient, which can effectively exploit the multilinear structure of tensor problems to develop lightweight updates. A tutorial of the `tensorlab`'s basic framework and updating rules can be found in (2). Users unfamiliar with tensors and nonlinear programming may also use `tensorlab` as a black box.

Selecting the Number of Communities

In principle, the number of Communities corresponds to the tensor rank used for minimizing the cost function in the main paper. The tensor rank is analogous to the number of principal components in the matrix principal component analysis (PCA) case. Due to noise and modeling error, the data tensors tend to have a high (or full) rank for real-life data. However, the "signal part" of the tensor is believed to have a low rank due to the high correlations across different modes.

We used $F = 6$ throughout the paper for several reasons. First, we hope to observe the most prominent migration systems and visualize their activities. Using $F = 6$ strikes a good balance between the visualization clarity and the migration system diversity. Second, we found that similar to matrix PCA, increasing the rank of the tensor model does not quite change the first 6 "principal components" (in our case, the first six rank-one tensors). This is generally not true for tensors due to the non-orthogonality of the latent factors (3). However, when one has nonnegative and (approximately) sparse latent factors, this property is often observed (4). For large-scale network analytics, the latent factors are often nonnegative and sparse. Therefore, we postulate that setting $F = 6$ yields the six most prominent migration systems under our model. Using $F > 6$ does not significantly change the first six migration systems compared to using $F = 6$.

We conduct the following experiment using the IRS data to verify our postulate. We gradually increase F and observe how this changes the first six rank-one terms. Let $\mathbf{A} \in \mathbb{R}^{I\times 6}, \mathbf{B} \in \mathbb{R}^{I\times 6}$ be the factors of decomposition with rank $F = 6$, and $\mathbf{A}' \in \mathbb{R}^{I\times F'}, \mathbf{B} \in \mathbb{R}^{I\times F'}$ be the factors of decomposition of the same input with rank $F' > 6$. The top 6 Communities are extracted and ordered for each decomposition based on their significance. Their similarity is then scored by

$$\texttt{Similarity score} = \frac{1}{12}\left(\sum_{i=1}^{6} \frac{|\text{top20}(\mathbf{a}_{\text{top-}i}) \cap \text{top20}(\mathbf{a}'_{\text{top-}i})|}{20} + \frac{|\text{top20}(\mathbf{b}_{\text{top-}i}) \cap \text{top20}(\mathbf{b}'_{\text{top-}i})|}{20} \right), \tag{2}$$

where $\mathbf{a}_{\text{top-}i}, \mathbf{b}_{\text{top-}i}$ denotes the columns in $\mathbf{A}, \mathbf{B}$ corresponding to the ith most significant Community, similarly for $\mathbf{a}'_{\text{top-}i}, \mathbf{b}'_{\text{top-}i}$, and $\text{top20}(\mathbf{x})$ denotes the index set of top 20 largest elements in $\mathbf{x}$. When the score

is close to 1, increasing the tensor rank does not change the top-20 counties/states in the top $-\ i$th most prominent migration system, for $i = 1, \ldots, 6$. As shown in Table. E.1, the top-6 extracted Communities using $F' = 7, \ldots, 19$ are all more than 65% similar to the case of using $F = 6$. In particular, when $F' \leq 11$, the similarity is above 72% in the worst case.

This supports our postulate.

Table E.1: The similarity of the top 6 extracted Communities for each $F > 6$ compared with $F = 6$.

Case study / F	7	8	9	10	11	12	13	14	15	16	17	18	19
MSA	0.8208	0.7750	0.7250	0.7292	0.7250	0.7583	0.6667	0.7542	0.6667	0.6792	0.6667	0.6042	0.6292
California	0.7708	0.7667	0.7917	0.7667	0.7667	0.6875	0.6750	0.6792	0.6792	0.7125	0.6875	0.6542	0.6500
Louisiana	0.8417	0.9042	0.8000	0.7250	0.7708	0.7542	0.7083	0.6875	0.7708	0.6917	0.6792	0.6750	0.6792

Comparison of 1990-2010 and 1990-2018

DeWaard et al. (5) discussed the potential pitfalls of using the combined 1990-2018 data due to a change in governmental agency post-processing. From 1990-2011, the post-processing was completed by the US Census Bureau. However, in 2012, the IRS started to post-process the IRS data internally. Because we employ a low-rank approximation to understand migration systems in the data, we do not *a priori* expect this change to be consequential for our analysis. Low-rank models explain the underlying essential correlations in the data and are thus often robust to changes in details. To quantify the impact of using such data, we perform the same decomposition procedure on two subsets of data to extract six Communities: a subset of data from 1990 to 2010 and the other from 1990 to 2018.

The result is again measured by the similarity score defined in the previous paragraph. In these cases, we compared the six Communities (1990-2011 and 1990-2018, respectively). We found that augmenting the data does not change the discovered prominent six migration systems significantly; see Table E.2. The slight change is reasonable since having data from more years is expected to offer more information—and the results should be different from only using the data from 1990-2011. Nonetheless, these relatively "stable" results also give us confidence that the difference in data post-processing does not damage our low-rank tensor model.

Case study	`Similarity score`
MSA	0.7125
All counties	0.7292
California	0.7583
Louisiana	0.7208

Table E.2: The `similarity score` of 6 Communities in 1990-2010 and 1990-2018.

References

[1] Vervliet, N., Debals, O., Sorber, L., Van Barel, M. & De Lathauwer, L. Tensorlab 3.0 (2016). URL `https://www.tensorlab.net`. Available online.

[2] Fu, X., Vervliet, N., De Lathauwer, L., Huang, K. & Gillis, N. Computing large-scale matrix and tensor decomposition with structured factors: A unified nonconvex optimization perspective. *IEEE Signal Processing Magazine* **37**, 78–94 (2020).

[3] Sidiropoulos, N. D. *et al.* Tensor decomposition for signal processing and machine learning. *IEEE Transactions on Signal Processing* **65**, 3551–3582 (2017).

[4] Papalexakis, E. E., Sidiropoulos, N. D. & Bro, R. From k-means to higher-way co-clustering: Multilinear decomposition with sparse latent factors. *IEEE Transactions on Signal Processing* **61**, 493–506 (2013).

[5] DeWaard, J. *et al.* User beware: Concerning findings from the post 2011–2012 us internal revenue service migration data. *Population Research and Policy Review* 1–12 (2021).
